# An integrated framework for agroclimatic, salinity, and groundwater assessment in the Jizzakh Region, Uzbekistan

**DOI:** 10.1016/j.isci.2026.117582

**Published:** 2026-10-06

**Authors:** B. Amanov, A. Ghirardelli, A. Sherov, A. Gafarova, D. Gulomov, P. Tarolli

**Affiliations:** 1 National Research University “Tashkent Institute of Irrigation and Agricultural Mechanization Engineering”, Tashkent, Uzbekistan; 2 Department of Land, Environment, Agriculture and Forestry, University of Padova, Padua, Italy

**Keywords:** agroclimatic indicators, water-salt balance, soil salinization, groundwater dynamics, climate change adaptation, Uzbekistan

## Abstract

Climate change, salinization, and water scarcity increasingly threaten irrigated agricultural landscapes in arid regions. This study developed an integrated framework combining hydro-climatic, soil, and groundwater indicators to assess agricultural landscapes in the Jizzakh Region, Uzbekistan. The framework integrated fourteen agroclimatic, soil-quality, and groundwater indicators into a unified scoring system. Results revealed persistent agroclimatic constraints during 2021–2024, with vegetation-period vapor pressure deficit (VPD) ranging from 1.91–1.37 kPa and hydrothermal coefficient (HTC) from 0.40–1.09, indicating high atmospheric water demand and semi-arid conditions. Groundwater mineralization remained predominantly within 1–3 g L^−1^, while salinity risks persisted in the areas affected by shallow groundwater and elevated mineralization. Integrated assessment scores ranged from 11 to 16 points, classifying most districts as *unsatisfactory* in 2021 and *satisfactory* during 2022–2024. Zafarabad and, occasionally, Sh. Rashidov exhibited the lowest overall scores. The framework provides a practical tool for monitoring landscape vulnerability and supporting irrigation, reclamation, and salinity-management decisions.

## Highlights

• Unified scoring system integrates fourteen climate, soil, and groundwater indicators
• Integrated assessment revealed spatial variability across irrigated landscapes
• Climate stress and shallow mineralized groundwater drive salinity risks
• Framework supports climate-resilient management in arid and semi-arid regions

## Graphical abstract

**Figure:**
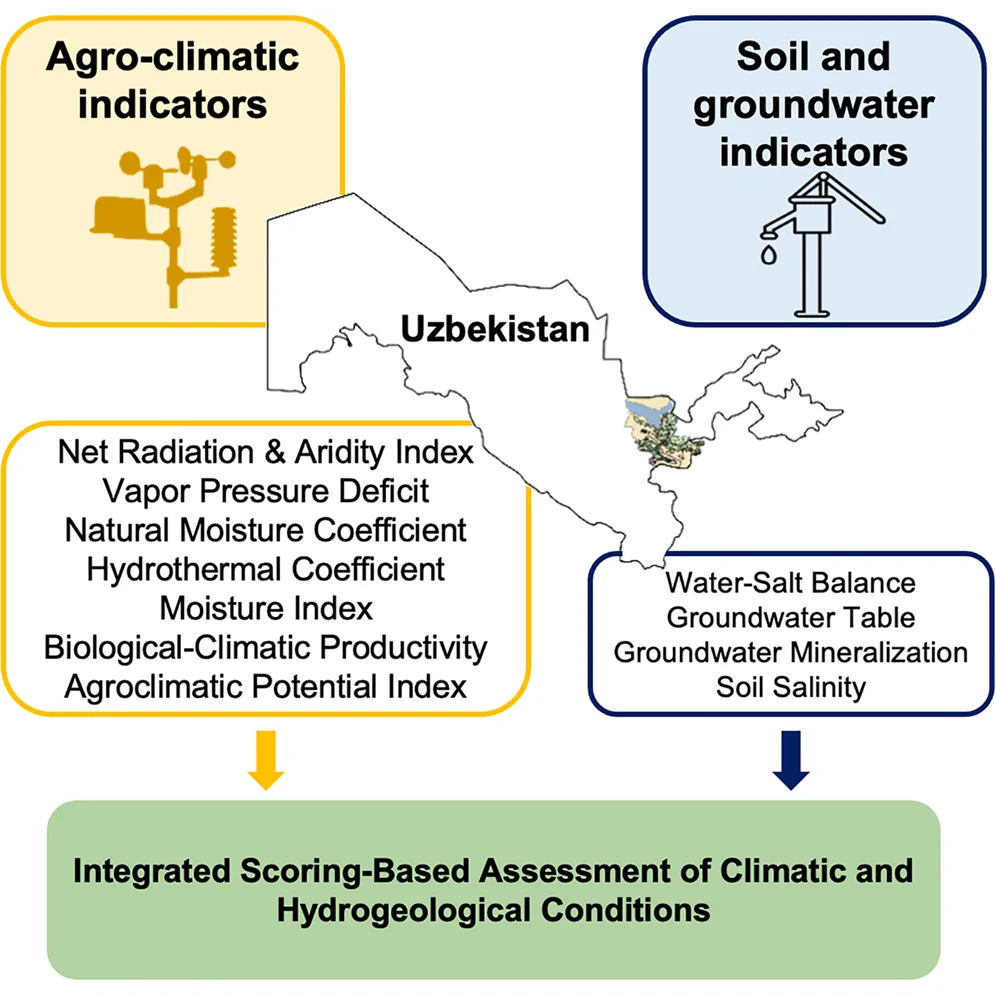

## Teaser

Soil science; Hydrology; Global change

## Introduction

Climate change, soil degradation, salinization, and the depletion of water resources pose serious and interconnected threats to the sustainability of agriculture and the stability of agricultural landscapes. Increasing air temperatures, greater evapotranspiration (ET), and altered precipitation regimes contribute to agricultural drought and the damaging of natural and agricultural ecosystems. Simultaneously, population growth and expanding agricultural production have intensified the extraction of surface and groundwater resources, resulting in progressive depletion and, in some areas, desertification. In irrigated landscapes, particularly in arid and semi-arid regions, inefficient irrigation systems and insufficient drainage have accelerated secondary salinization processes, reducing soil fertility, disturbing hydrological balance, and limiting crop productivity.^1^ These combined factors lead to a deterioration of soil structure, nutrient depletion, and loss of biodiversity, ultimately threatening the long-term resilience of agroecosystems and the livelihoods that depend on them.

In Uzbekistan, these challenges are intensified by both natural and anthropogenic factors, including erosion, salinization, and inefficient water use. According to the UN Convention to Combat Desertification, approximately 28.6% of Uzbekistan’s territory is affected by land degradation.^2^ Recent Food and Agriculture Organization (FAO) reports note significant restoration efforts, including the recovery of 13,000 hectares of degraded land and the implementation of climate adaptation measures on up to 225,000 hectares of agricultural land.^3^ These pressures are particularly evident in the irrigated landscapes of the Jizzakh Region (Figure 1), where agricultural production depends heavily on irrigation and is increasingly influenced by climatic aridity, groundwater dynamics, and soil salinization. Consequently, reliable methods for assessing the combined effects of climatic, soil, and hydrogeological factors are essential for sustainable land and water management and ultimately food security.

**Figure 1:**
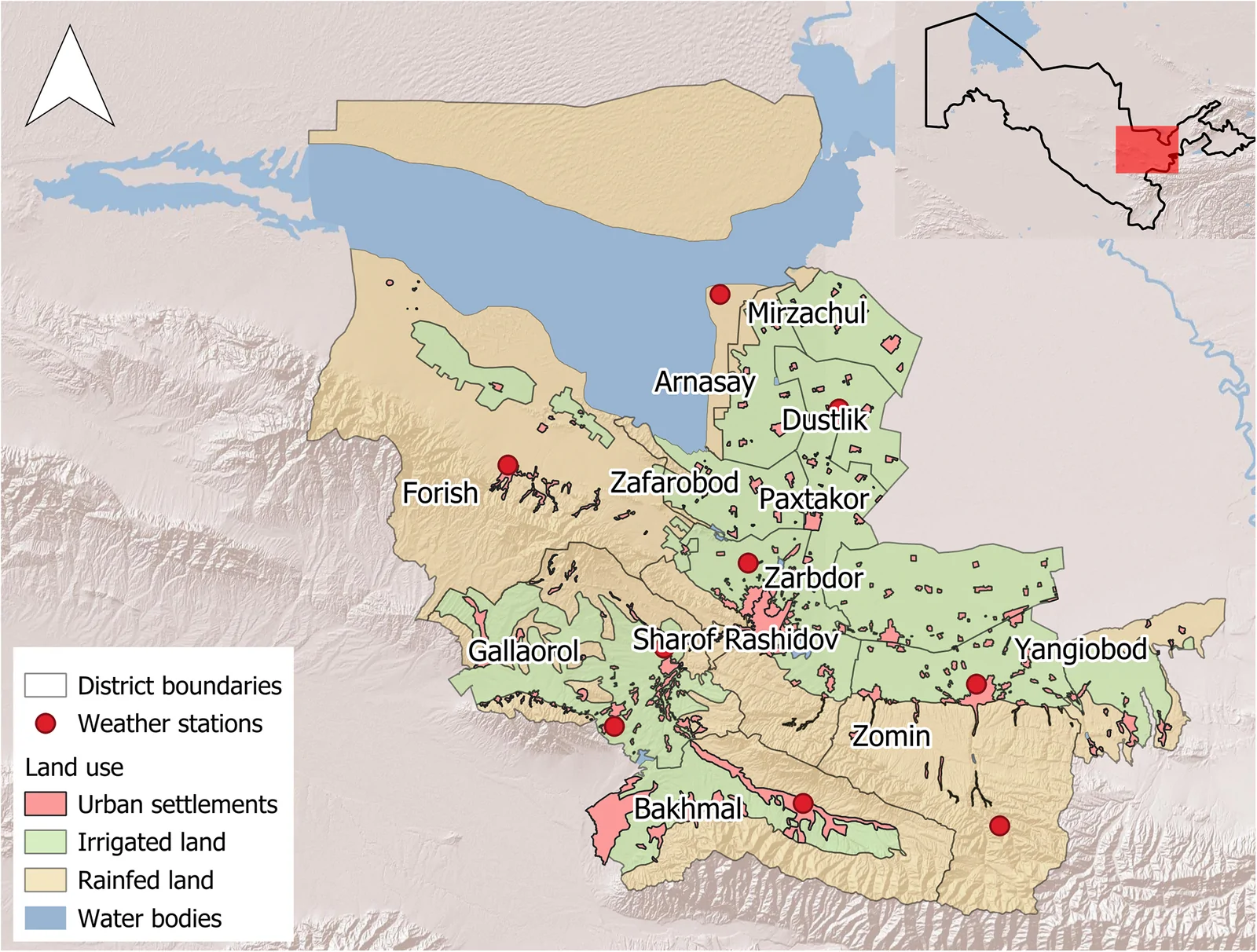
Study Area and Meteorological Stations in Jizzakh Region, Uzbekistan Different colors indicate land-use types across the region’s districts.

Comprehensive assessment of agricultural landscapes using climatic indicators is essential for understanding current conditions, optimizing resource use, and identifying opportunities for adaptation. Previous research has consistently highlighted the importance of integrating climatic, soil, and groundwater indicators for the sustainable management of irrigated landscapes. Studies have shown that systematic monitoring and objective evaluation of climatic factors, including radiation balance, vapor pressure deficit (VPD), aridity indices, and humidity and hydrothermal coefficients (HTCs), are critical for understanding the impacts of climate variability on productivity and landscape stability.^4^^,^^5^ Agroclimatic metrics such as biological-climatic productivity (BCP) and agroclimatic potential index (ACPI) have been applied to quantify the effects of temperature and precipitation regimes on agricultural outputs and ecosystem functions.^6^^,^^7^ In parallel, monitoring of soil and water conditions, including groundwater table depth, soil salinity, and degree of soil salinization, has proven essential for evaluating land-reclamation needs and irrigation efficiency.

While numerous studies address subsets of these indicators, such as climate-driven soil salinization, mapping of salt-affected soils,^1^^,^^8^ or groundwater salinity,^9^ few integrate all components into a unified, landscape-scale assessment framework. Soil-quality indices that combine multiple chemical and physical parameters,^10^^,^^11^ demonstrate the value of composite scoring systems for assessing land condition and guiding sustainable management; however, they often focus primarily on soil properties and may omit climatic dynamics, groundwater conditions, or continuous field-monitoring data. Similarly, hydrological models that account for water-salt balance provide detailed insights into irrigation impacts but rarely link these results to agroclimatic productivity metrics or landscape-scale evaluations.^12^ Thus, there is a clear gap in the literature for an integrated assessment framework that combines continuous meteorological observations, calculated climatic indices, BCP metrics, groundwater depth and salinity measurements from monitoring wells, and derived soil-quality indicators into a single, comprehensive scoring system. This is particularly relevant in Central Asia, where irrigated agriculture is highly vulnerable to salinization and water scarcity, yet integrated assessment frameworks remain limited.^13^ Additionally, existing approaches often require complex datasets that are difficult to compare across districts and management units and approachable for decision makers. As a result, decision-makers frequently lack a practical tool capable of synthesizing climatic, soil, groundwater, and reclamation information into an overall assessment of landscape conditions.

Understanding the interactions between agriculture, biodiversity, and climate has become one of the most urgent global research priorities. Climate change affects these interactions by altering ecosystem structure and function, with consequences for agricultural productivity.^4^ In recent years, attention has focused on the meteorological and hydrological factors that shape agricultural productivity. Foundational studies^7^^,^^14^ emphasize the importance of geographic zonality, energy balance, and hydrothermal regimes. Commonly applied indices include the Budyko radiation aridity index, which expresses the precipitation-evaporation balance,^15^^,^^16^ the De Martonne aridity index, a simple yet robust climate classification metric,^17^ and Selyaninov’s HTC, which characterizes drought intensity during the growing season.^18^^,^^19^ These indicators provide a solid foundation for regional and temporal analysis under the climatic conditions of Uzbekistan, where agroclimatic resources, water-salt balance, salinization, and drainage status are under increasing pressure from climate change and intensive irrigation practices. Periodic assessment of current and projected climate impacts is therefore vital for updating natural resource management plans and evaluating adaptation measures,^5^ while supporting the long-term sustainability and resilience of irrigated cropland.

To address this need, the present study develops a comprehensive, integrated scoring framework for the assessment of irrigated lands and landscapes in the Jizzakh Region, Uzbekistan. This framework combines climatic, soil, and groundwater quality indicators to provide an objective and multidimensional evaluation of landscape status. Climatic indicators include radiation balance, VPD, aridity and humidity coefficients, HTCs, and BCP alongside ACPI, capturing both water and energy constraints on agricultural production. Soil and groundwater quality indicators, derived from field-based monitoring wells, encompass groundwater depth, soil salinity, and the degree of soil salinization, thereby reflecting land-reclamation needs and irrigation efficiency. Unlike conventional approaches that evaluate these indicators separately, the proposed methodology integrates them within a unified scoring framework that enables direct comparison and aggregation of multiple environmental factors. The resulting three-level classification system is designed to be transparent, easy to interpret, and readily applicable using data routinely collected through existing meteorological, groundwater monitoring, and land-reclamation programs. This approach facilitates rapid, comparative assessments across farms and districts, supports evidence-based resource management, and provides early warning for areas at risk of degradation. Moreover, by linking climate variability, soil and groundwater status, and agroclimatic productivity, the system enables the identification of adaptation opportunities and the design of targeted interventions. In doing so, the study not only addresses a critical regional knowledge gap but also contributes to the development of a methodology that can be adapted for integrated landscape assessment in other arid and semi-arid regions. The resulting framework provides a scientifically robust and practically applicable tool to support sustainable agriculture under the dual pressures of climate change and water scarcity.

## Results

### Assessment of agroclimatic indicators

Spatial differentiation of the radiation balance across the Jizzakh Region provides a quantitative basis for agroecological zoning and for planning differentiated irrigation and cropping strategies. Tables 1 and S1 present the net radiation (*R*) calculated for each district during non-vegetation, vegetation, and annual periods for both total and irrigated areas. During 2021–2024, the *R* value for irrigated lands in districts represented by the Dustlik meteorological station ranged from 306 to 311 kJ cm^−2^ month^−1^, while values for total areas were slightly lower, averaging 298–305 kJ cm^−2^ month^−1^. These results indicate a consistently higher energy input and evaporative demand in irrigated zones compared with non-irrigated areas. In contrast, districts associated with the Jizzakh meteorological station showed generally lower radiation balance values, 271–351 kJ cm^−2^ month^−1^ for total areas and 280–359 kJ cm^−2^ month^−1^ for irrigated areas. Notably, in 2024, several districts recorded a marked increase in radiation balance (up to 358–359 kJ cm^−2^ month^−1^), reflecting elevated agroclimatic energy and ET potential. Overall, the spatial pattern of radiation balance follows the plain-piedmont-mountain gradient characteristic of the region’s agricultural landscapes, with values decreasing from west to east and from plains to uplands. Districts such as Arnasay, Dustlik, and Paxtakor exhibited the highest radiation fluxes and thus the greatest irrigation requirements, while Zomin, Zarbdor, and Sh. Rashidov displayed lower radiation inputs associated with higher elevation and orographic effects.

To account for irrigation effects, aridity indices were calculated both with the classic Budyko’s equation ($\bar{R}$, Tables 1 and S2) and separately modified equations for total and irrigated areas (${\bar{R}}_{UM}$ and ${\bar{R}}_{CM}$, Tables 1 and S3). This distinction allows evaluation of how irrigated agriculture mitigates drought stress and influences the overall water-energy balance. The results show that the aridity index remained relatively stable from 2021 to 2024 in the districts of Sh. Rashidov, Zarbdor, and Zomin, reflecting consistent precipitation and limited interannual variability. Across other districts, the index fluctuated only slightly, consistent with the generally uniform meteorological conditions observed during the study period.

**Table 1:** District-level net radiation (*R*, kJ cm^−2^ month^−1^) and aridity index with classic ($\bar{R}$) and modified (${\bar{R}}_{UM}$ and ${\bar{R}}_{CM}$) Budyko’s equation for total and irrigated areas in Jizzakh Region, years 2021–2024

Year	District	*R* (kJ cm^−2^ month^−1^)		$\bar{R}$		${\bar{R}}_{UM}$	${\bar{R}}_{CM}$
		Total area	Irrig. area	Total area	Irrigated area	Total area	Irrigated area
2021	Arnasay	303	308	0.47	0.48	1.83	1.43
2022		304	310	0.29	0.30	1.41	1.14
2023		301	307	0.45	0.46	1.85	1.16
2024		305	311	0.33	0.34	1.64	1.26
2021	Dustlik	302	308	0.47	0.48	1.26	1.36
2022		300	306	0.29	0.29	1.29	1.36
2023		300	307	0.45	0.46	1.41	1.71
2024		305	311	0.33	0.34	1.50	1.77
2021	Zafarabad	300	308	0.47	0.48	1.44	1.55
2022		298	306	0.29	0.29	1.30	1.45
2023		298	307	0.44	0.46	1.45	1.51
2024		303	311	0.33	0.34	1.44	1.56
2021	Mirzachul	301	308	0.47	0.48	1.27	1.50
2022		299	306	0.29	0.29	1.36	1.45
2023		301	306	0.45	0.46	1.29	1.45
2024		304	311	0.33	0.34	1.21	1.46
2021	Paxtakor	302	308	0.47	0.48	1.37	1.49
2022		299	306	0.29	0.29	0.99	1.08
2023		300	307	0.45	0.46	1.55	1.38
2024		305	311	0.33	0.34	1.50	1.37
2021	Sh.Rashidov	281	288	0.43	0.44	1.23	1.48
2022		273	280	0.21	0.21	1.04	1.41
2023		287	293	0.37	0.38	1.21	1.26
2024		311	358	0.38	0.39	1.37	1.50
2021	Zarbdor	281	288	0.43	0.44	1.20	1.27
2022		273	280	0.21	0.21	0.99	1.12
2023	–	286	293	0.37	0.38	1.38	1.27
2024		350	358	0.38	0.39	1.57	1.54
2021	Zomin	278	288	0.43	0.44	1.17	2.79
2022		271	280	0.20	0.21	1.02	3.44
2023		283	293	0.37	0.38	1.29	2.76
2024		346	359	0.38	0.39	1.44	2.81

The long-term variability of VPD was evaluated using data from the Jizzakh and Dustlik meteorological stations for vegetation, non-vegetation, and total annual periods (Table 2). Analysis of these data indicates that during the vegetation period, long-term average VPD reached 1.48 kPa at Dustlik and 1.60 kPa at Jizzakh, reflecting severe water stress conditions for crops. In contrast, VPD during the non-vegetation period remained low (0.25 kPa), while total long-term averages of 0.97 kPa (Dustlik) and 1.04 kPa (Jizzakh) underscore the arid nature of the region.

**Table 2:** Vapor pressure deficit (kPa) for Jizzakh and Dustlik meteorological stations, 2021–2024 and long-term average

Meteorological station	Period	2021	2022	2023	2024	Long-term average
Jizzakh	non-vegetation	0.28	0.24	0.33	0.27	0.25 (88 years)
	vegetation	1.79	1.57	1.71	1.65	1.60 (88 years)
	total	1.16	1.01	1.13	1.07	1.04 (88 years)
Dustlik	non-vegetation	0.36	0.25	0.33	0.21	0.25 (52 years)
	vegetation	1.91	1.51	1.79	1.37	1.48 (52 years)
	total	1.27	0.99	1.17	0.89	0.97 (52 years)

Analysis of moisture coefficient (*C*_*m*_) data from the Jizzakh and Dustlik meteorological stations indicates that *C*_*m*_ in both regions has remained below long-term averages over the past 3 years (Table 3). The Jizzakh station exhibits a clear aridification pattern, whereas Dustlik has maintained relatively better conditions, although water scarcity persists. During 2021–2024, the overall *C*_*m*_ at both stations remained consistently below the long-term averages, indicating increasing moisture deficiency and a trend toward aridity. The 88-year average for Jizzakh is 0.29, and the 52-year average for Dustlik is 0.26, whereas recent annual values ranged from 0.13 to 0.47, consistently remaining below 1, which is considered low. Seasonal variations reveal that in the non-vegetation period, the *C*_*m*_ is relatively high (averaging 1.27 for both Jizzakh and Dustlik), reflecting adequate natural water availability during this season. In contrast, during the vegetation period, *C*_*m*_ is extremely low (averaging 0.12 in Jizzakh and 0.10 in Dustlik), indicating acute water deficiency for crops and highlighting the significance of drought-related risks.

**Table 3:** Natural moisture coefficient (*C*_*m*_) at Jizzakh and Dustlik meteorological stations, 2021–2024 and long-term averages

Meteorological station	Period	2021	2022	2023	2024	Long-term average
Jizzakh	non-vegetation	1.01	2.12	1.09	1.56	1.27 (88 years)
	vegetation	0.03	0.22	0.05	0.13	0.12 (88 years)
	total	0.16	0.47	0.20	0.31	0.29 (88 years)
Dustlik	non-vegetation	0.74	2.10	0.86	1.70	1.27 (52 years)
	vegetation	0.02	0.11	0.05	0.14	0.10 (52 years)
	total	0.13	0.34	0.16	0.33	0.26 (52 years)

The spatial and temporal variability of moisture index (*I*_*m*_) provides valuable insights into the hydrothermal conditions of the Jizzakh Region’s agricultural landscapes, encompassing plain, piedmont, and alluvial zones (Table 4). *I*_*m*_ values varied substantially between years. The highest values were observed in 2022 (1.31 in Jizzakh and 1.16 in Dustlik), corresponding to increased precipitation, while 2023 showed a marked decline (0.56 and 0.49), indicating drier conditions. During the vegetation period, values remained consistently low (0.01–0.10), reflecting acute moisture deficits and confirming the semi-arid character of the region. Long-term averages (annual $I_{m}$ = 0.13 for Jizzakh and 0.12 for Dustlik) indicate persistent aridity, with only temporary improvements during wetter years.

**Table 4:** Moisture index ($I_{m})$ at Jizzakh and Dustlik meteorological stations, 2021–2024 and long-term averages

Meteorological station	Periods	2021	2022	2023	2024	Long-term average
Jizzakh	non-vegetation	0.60	1.31	0.56	0.85	0.87 (88 years)
	vegetation	0.01	0.10	0.02	0.06	0.05 (88 years)
	total	0.07	0.21	0.09	0.14	0.13 (88 years)
Dustlik	non-vegetation	0.43	1.16	0.49	1.03	0.75 (52 years)
	vegetation	0.01	0.06	0.02	0.07	0.05 (52 years)
	total	0.06	0.17	0.07	0.17	0.12 (52 years)

The HTC reflects the balance between heat and moisture during the growing season and serves as an effective indicator for assessing regional hydrothermal conditions and agricultural potential. In this study, HTC was calculated for the Jizzakh and Dustlik meteorological stations to evaluate recent climatic dynamics and their implications for agricultural productivity (Table S4). During 2021–2024, the HTC fluctuated notably at both meteorological stations, reflecting strong interannual climate variability. The highest values were observed in 2022, 0.95 in Dustlik and 1.09 in Jizzakh, corresponding to increased precipitation and improved moisture conditions for crops. In contrast, 2021 and 2023 exhibited much lower values (0.4–0.5), indicative of arid conditions and elevated irrigation demand. By 2024, HTC partially recovered (0.84 in Dustlik and 0.77 in Jizzakh), suggesting a return to moderately humid conditions.

The BCP index was evaluated for the Jizzakh Region under Central Asian climatic conditions, using data from the Jizzakh and Dustlik meteorological stations. Calculations were conducted separately for the vegetation and non-vegetation periods, incorporating HTC. The results are summarized in Table 5, which presents mean BCP values, seasonal variability, and long-term averages for both meteorological stations. During 2021–2024, BCP values exhibited strong interannual and spatial variability. At the Jizzakh station, total BCP ranged from 0.8 in 2021 to 2.43 in 2022, before decreasing to 1.04 in 2023 and partially recovering in 2024. The long-term 88-year average was 1.42, illustrating pronounced climatic instability. The highest BCP values in 2022 corresponded to a year of above-average precipitation (596.1 mm), like anomalously wet years such as 1953 and 1993. At the Dustlik station, total BCP varied between 0.66 in 2021 and 1.70 in 2022, with a long-term 52-year mean of 1.25. The non-vegetation periods of 2023–2024 showed relatively high BCP values compared to the vegetation season, underscoring the seasonal dependence of productivity on water availability. As at Jizzakh, the 2022 peak coincided with elevated precipitation (469 mm), a pattern comparable to wet years in 1993 and 1998.

**Table 5:** Biological-climatic productivity values for the Jizzakh and Dustlik meteorological stations for 2021–2024, compared with long-term averages

Meteorological station	Periods	2021	2022	2023	2024	Long-term average
Jizzakh	non-vegetation	0.38	0.67	0.33	1.34	0.37 (88 years)
	vegetation	0.14	1.10	0.25	0.66	0.54 (88 years)
	total	0.80	2.43	1.04	1.78	1.42 (88 years)
Dustlik	non-vegetation	0.00	0.00	0.71	0.29	0.20 (52 years)
	vegetation	0.12	0.55	0.23	0.67	0.48 (52 years)
	total	0.66	1.70	0.94	1.60	1.25 (52 years)

Values are presented for vegetation, non-vegetation, and annual periods.

To complement these findings, a land-quality-based classification system was applied to evaluate productivity potential according to soil fertility categories (Table 6). This system integrates soil bonitet scores, defined according to national Uzbek regulations,^20^^,^^21^ with biological productivity indicators. Table 6 shows that in *poor lands* (bonitet <20), BCP values are around 0.63, indicating low fertility and weak agrotechnical performance; these areas require repeated reclamation and fertility restoration measures. In *below-average lands* (21–40 points), BCP ranges from 0.66 to 1.26, reflecting modest productivity gains achievable through additional soil improvement practices. *Average lands* (41–60 points) exhibit BCP values of 1.29–1.89, with moderate fertility and good potential for efficiency enhancement. *Good lands* (61–80 points) show BCP between 1.92 and 2.32, corresponding to high soil fertility and well-developed agronomical management, while *best lands* (81–100 points) attain BCP values of 2.55–3.15, indicating maximum productivity and minimal yield gaps (Y_**actual**_/Y_**max**_ ≈ 0.8–1.0).

**Table 6:** Classification of agricultural land productivity based on meliorative soil bonitet (*B*_*m*_) scores and corresponding BCP, maximum potential productivity (*B*_*max*_), and actual-to-maximum yield ratio (Y_actual_/Y_max_)

Score categories^a^	Soil bonitet score (or soil bonitet)^b^	*B*_*m*_	*B*_*mах*_	Y_actual_/Y_max_
Poor lands	˂20	0.63	2.43	0.2
Below-average lands	21–40	0.66÷1.26		0.21÷0.4
Average lands	41–60	1.29÷1.89		0.41÷0.6
Good lands	61–80	1.92÷2.32		0.6÷0.80
Best lands	81–100	2.55÷3.15		0.81÷1

Categories follow the national soil bonitet standards for irrigated lands.

a In this table, the indicator is classified according to the soil bonitet score categories of irrigated lands.

b Based on the requirements set by the Decree of the President of the Republic of Uzbekistan no. PF-4478 dated October 22, 2012, and the Resolution of the Cabinet of Ministers no. 235, a system for improving the determination of the normative value of agricultural crop areas has been developed. Within this framework, normative productivity indicators per soil bonitet score have been established.

Annual and long-term ACPI values for the Jizzakh and Dustlik meteorological stations are presented in Table 7. ACPI values at both stations range from 0.04 to 0.11, with long-term averages between 0.06 and 0.08. When interpreted using the Kazakhstan classification proposed by Mustafayev and Ryabtsev,^22^ where forest-steppe zones reach 0.13–0.17, steppes reach 0.15, semi-deserts reach around 0.10, and northern and southern deserts reach 0.08 and 0.07, respectively, the Jizzakh Region clearly falls within the desert to semi-desert categories. The comparatively higher ACPI values recorded in 2022 (0.11 in Jizzakh and 0.09 in Dustlik) correspond to a year with increased precipitation, temporarily improving agroclimatic conditions.

**Table 7:** Agroclimatic potential index values for the Jizzakh and Dustlik meteorological stations

Meteorological station	2021	2022	2023	2024	Long-term average
Jizzakh	0.04	0.11	0.05	0.08	0.08 (88 years)
Dustlik	0.04	0.09	0.05	0.08	0.06 (52 years)

### Assessment of soil and groundwater indicators

Groundwater depths during 2021–2024 varied significantly among districts (Figure 2). In Arnasay and Dustlik, the groundwater depth showed a slight increase compared to earlier years, suggesting improved drainage conditions or reduced recharge. Zafarabad also exhibited a more noticeable deepening of the groundwater table. In contrast, Zarbdor and Paxtakor districts recorded decreasing groundwater depths, while Sh. Rashidov district displayed a consistent deepening trend throughout the study period. Across the districts of Jizzakh Region, groundwater mineralization during the vegetation period predominantly falls within the 1–3 g/L range, forming the largest share in every district (Figure 3). In Dustlik and Sh. Rashidov districts, this class accounts for nearly 90% of the monitored area. Low-mineralized groundwater (0–1 g/L) occurs sporadically but has expanded in several districts, such as Dustlik, Zarbdor, Zafarabad, Paxtakor, Sh. Rashidov, and Zomin, in recent years. By contrast, areas with moderately mineralized groundwater (3–5 g/L) have increased in Arnasay, Dustlik, Zafarabad, and Paxtakor by 2023 and subsequently in Zarbdor and Mirzachul by 2024.

**Figure 2:**
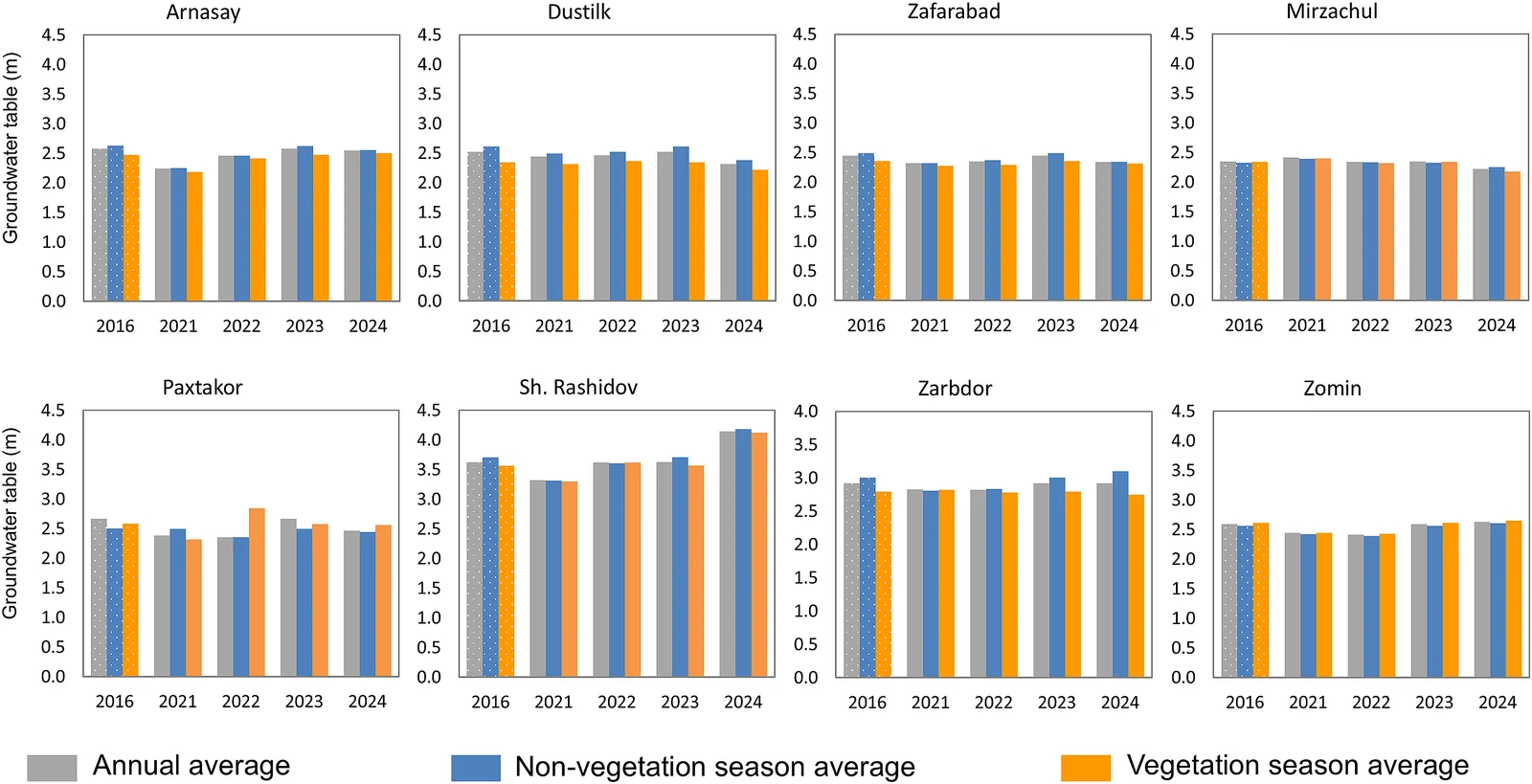
Groundwater table depth by districts of Jizzakh Region Bars show annual average, vegetation-period, and non-vegetation-period groundwater depths in each district. Data for 2016 are included as a reference baseline for comparison with the 2021–2024 monitoring period.

**Figure 3:**
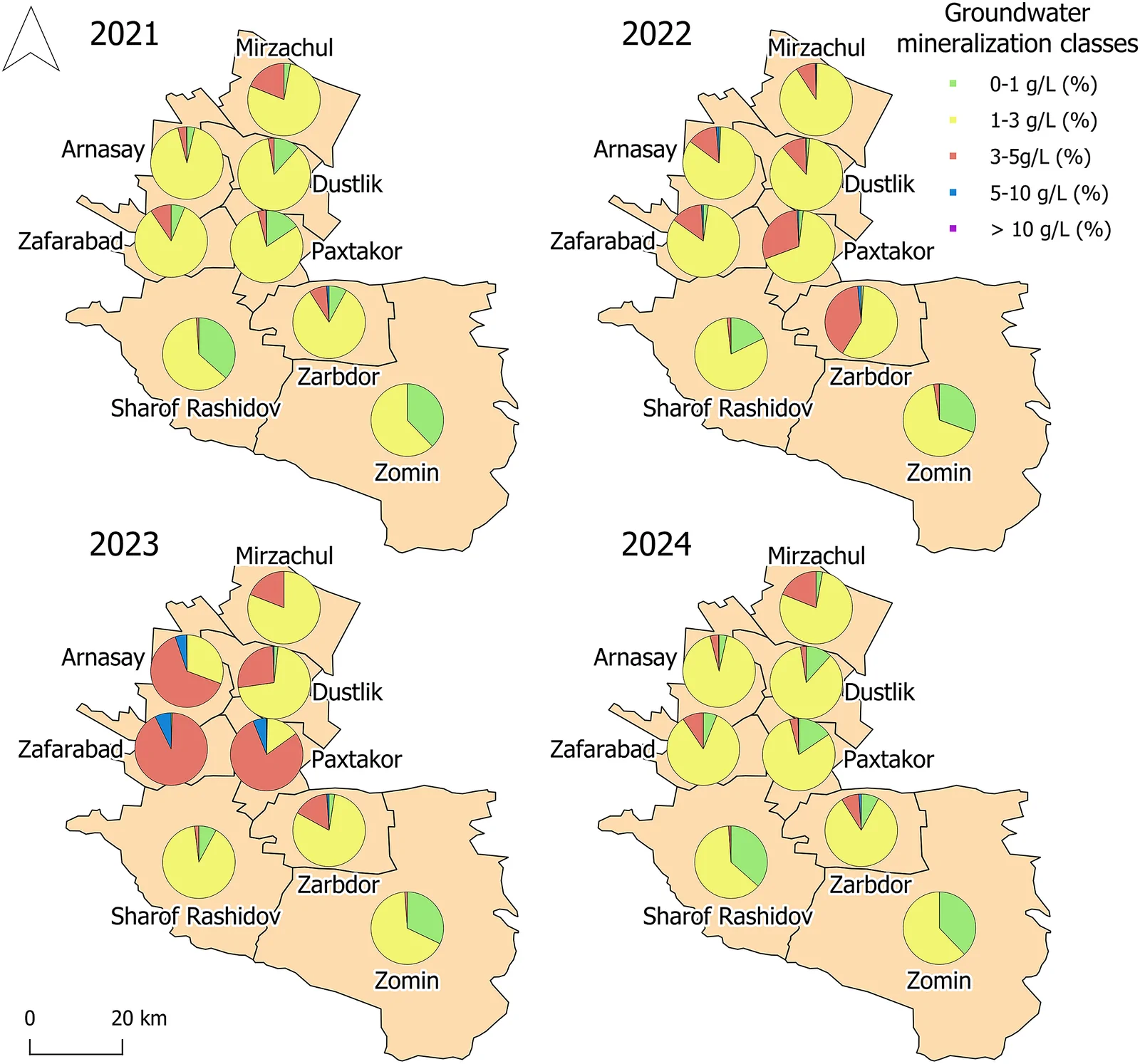
Distribution of groundwater mineralization by district during the vegetation period in the Jizzakh Region (2021–2024) Maps show the percentage distribution of groundwater mineralization classes in each district during the vegetation period for 2021–2024.

Soil salinity determined in autumn, at the end of vegetation season, shows that low and moderate salinity predominate across most districts (Figure 4). In recent years, the share of non-saline areas has increased in Dustlik, Zafarabad, Mirzachul, and Arnasoy, though in Arnasoy and Mirzachul non-saline land remains limited (1.4%–6.5%). Slightly saline areas have increased in Zafarabad and Zomin, while in Arnasoy, Mirzachul, Dustlik, and Paxtakor they have fluctuated. Moderately saline soils have increased to 1%–5% in Arnasoy, Zarbdor, Zomin, Paxtakor, and Sh. Rashidov, but decreased in Dustlik and Zafarabad. Strongly and very strongly saline areas remain small and concentrated in Paxtakor, Zarbdor, Dustlik, Zafarabad, Arnasoy, and Mirzachul, with an overall decreasing trend from 2021 to 2024. In 2023, 73% of irrigated lands exhibited some degree of salinity, slightly declining to 71.4% in 2024. Historical data indicate that in 2011, 79% of irrigated lands were affected by salinity, with 56.6% slightly saline and 15% moderately saline. Analysis of the water-salt balance inputs for 2021–2024 (Table S5) indicates significant variability across districts in both irrigation water and precipitation. For instance, irrigation volumes ranged from 6,538 m^3^/ha in Zomin (2023) to 11,071 m^3^/ha in Paxtakor (2022), reflecting differences in crop water demand, soil moisture deficits, and local irrigation management. Salt input through irrigation also varied, with the highest contributions observed in Zafarabad (12.24 t/ha in 2021) and Paxtakor (11.19 t/ha in 2021). The output components of the water-salt balance, including ET, drainage, and salt removal (Table S6), further elucidate the effectiveness of water use and the state of soil reclamation. ET values ranged from 6,274 m^3^/ha (Dustlik, 2023) to 8,929 m^3^/ha (Paxtakor, 2024), highlighting crop water requirements and climatic influence. Drainage volumes exhibited substantial interannual variability, with high values in Mirzachul (5,600 m^3^/ha in 2021) and Paxtakor (6,746 m^3^/ha in 2022), which are crucial for removing excess water and salts from the root zone. Salt removal values were highest in Zafarabad (3.46 t/ha, 2021), Paxtakor (3.06 t/ha, 2021), and Dustlik (2.39 t/ha, 2023), reflecting effective leaching and reduced risk of soil salinization. Total annual water outflow (ET + drainage) ranged from 12,000 to 15,500 m^3^/ha, while total salt removal varied between 1.5 and 3.5 t/ha.

**Figure 4:**
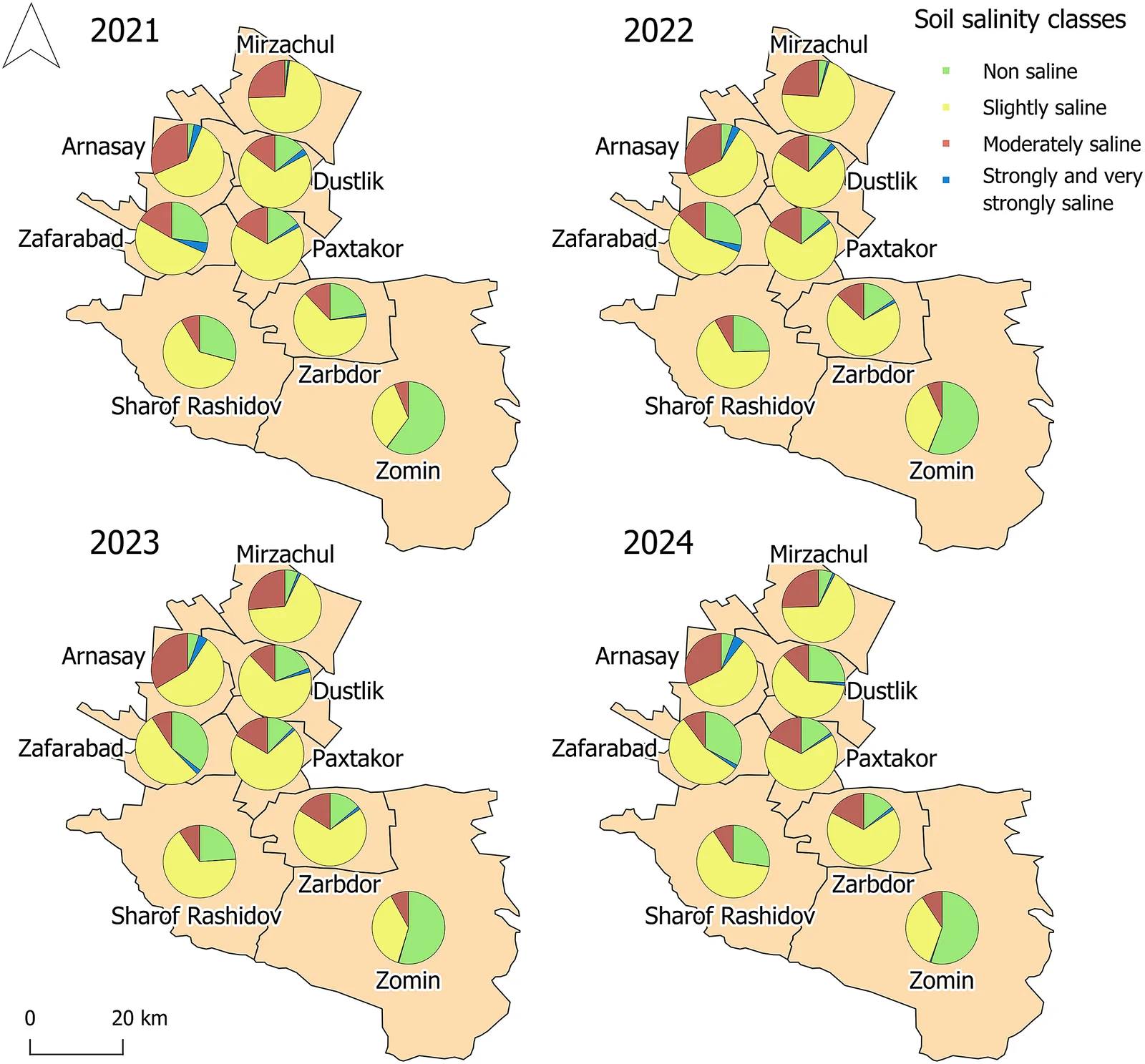
Annual distribution of areas with different soil salinity levels in Jizzakh Region based on autumn measurements Maps show the percentage distribution of soil salinity classes within each district for 2021–2024. Classes are based on the dry residue of soluble salts and chlorine content in the 0–100 cm soil layer.

The 2021–2024 analysis of overall water and salt balances (Table 8) shows that most districts experience a negative total water balance, indicating water deficits. For example, Arnasay (2021), Zafarabad (2021), and Zomin (2022) had a deficit of 2,807, 2,060, and 2,692 m^3^/ha, respectively. During the vegetation period, negative balances highlight water scarcity for crops, whereas positive balances in the non-vegetation period (observed in Dustlik, Zarbdor, Mirzachul, and Paxtakor) indicate restoration of water reserves. The total salt balance is generally positive in most districts, indicating a risk of soil salinization (e.g., 2.43 t/ha in Zafarabad, 2021; 6.48 t/ha in Sh. Rashidov, 2023; and 4.3 t/ha in Paxtakor, 2021). In contrast, negative salt balances in certain districts and years (e.g., −5.1 t/ha in Zomin, 2022 and −1.29 t/ha in Paxtakor, 2022) indicate active salt leaching, which is beneficial for maintaining soil fertility. Positive salt balances during the vegetation period, particularly in Arnasay, Zafarabad, Paxtakor, and Sh. Rashidov, are a critical risk factor for soil salinity.

**Table 8:** Overall water and salt balance in the districts of Jizzakh Region (2021–2024), indicating net water and salt gains or deficits during vegetation and non-vegetation periods

District	Years	Water balance (m^3^/ha)			Salt balance (t/ha)		
		Total	Vegetation	Non-vegetation	Total	Vegetation	Non-vegetation
Arnasay	2021	−2,807	−3,099	292	0.48	3.01	−2.53
	2022	−1,324	−2,358	1,035	1.68	2.37	−0.69
	2023	−3,013	−3,159	146	2.69	4.68	−1.99
	2024	−2,152	−2,193	42	1.92	3.37	−1.45
Dustlik	2021	−404	−3,223	2,819	0.68	0.67	0.01
	2022	65	−1,368	1,433	0.99	1.72	−0.73
	2023	436	−1,115	1,551	0.68	1.08	−0.4
	2024	273	−764	1,036	0.17	0.75	−0.58
Zafarabad	2021	−2,060	−3,278	1,217	2.43	2.05	0.38
	2022	−1,249	−2,370	1,121	0.06	1.62	−1.56
	2023	−2,074	−2,832	757	1.07	3.9	−2.83
	2024	−1,416	−1,637	222	0.95	3.15	−2.2
Mirzachul	2021	−842	−3,249	2,407	−1.24	0.36	−1.61
	2022	−347	−1,860	1,513	0.47	0.95	−0.48
	2023	−244	−1,182	938	1.04	2.47	−1.43
	2024	220	−999	1,219	−0.74	0.47	−1.2
Paxtakor	2021	−335	−1,537	1,202	4.3	4.5	−0.2
	2022	−275	−3,154	2,879	−1.97	−0.68	−1.29
	2023	−2,379	−3,014	635	0.58	3.02	−2.44
	2024	−1,903	−1,969	65	0.66	2.82	−2.17
Sh.Rashidov	2021	−485	−2,225	1,740	2.79	2.9	−0.11
	2022	−556	−1,696	1,140	−0.92	0.23	−1.16
	2023	−636	−1,746	1,110	6.48	4.85	1.62
	2024	27	−787	814	6.5	4.09	2.41
Zarbdor	2021	194	−1,799	1,993	3.1	2.14	0.95
	2022	501	−932	1,433	1.21	−0.44	1.64
	2023	−789	−1,590	801	3.74	3.95	−0.21
	2024	−361	−946	584	3.82	3.04	0.78
Zomin	2021	−1,102	−2,627	1,526	1.21	1.66	−0.45
	2022	−2,692	−2,265	−427	−8.39	−3.29	−5.1
	2023	−1,135	−1,850	715	1.35	2.31	−0.96
	2024	−277	−905	629	1.8	2.04	−0.25

### Comprehensive assessment of the agroclimatic and hydrogeological state

The developed methodology integrates the principal factors governing land productivity and sustainability, water-salt balance, groundwater depth and mineralization, soil salinity levels, agroclimatic indices, and biological productivity, into a unified quantitative framework (Table S7). By combining hydrological, soil, and agroclimatic indicators, the scoring system provides an aggregated measure of landscape condition and vulnerability (Figure 5). The integrated assessment yielded total scores ranging from 11 to 16 points across the studied districts during 2021–2024. The majority attained *satisfactory* status during 2022–2024, while most districts were classified as *unsatisfactory* in 2021. However, total scores generally remained within the lower to middle portion of the satisfactory range (14–16 points), indicating that favorable hydrological and agronomical conditions were not consistently achieved across all evaluated indicators.

**Figure 5:**
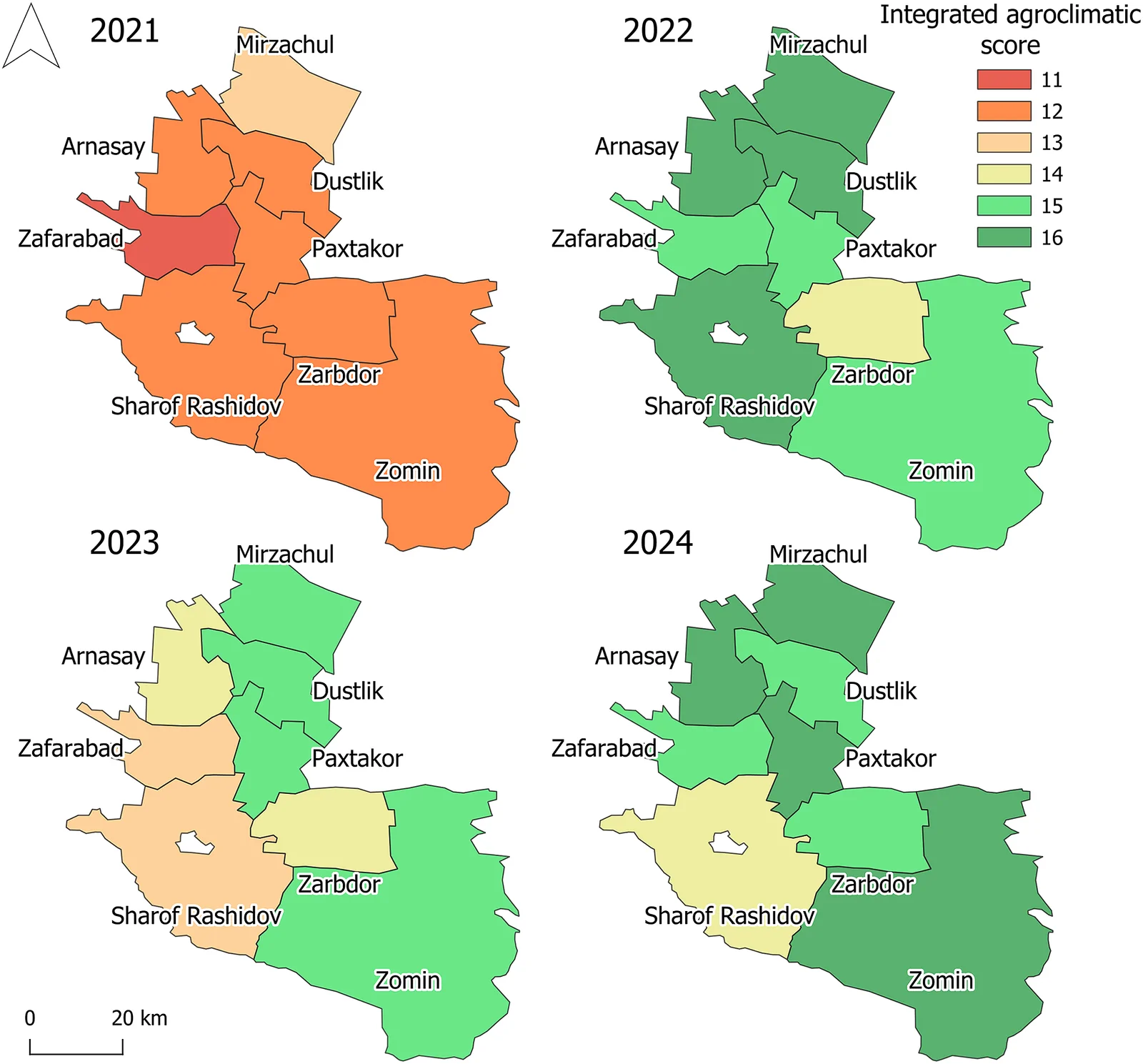
Spatial patterns of district-level integrated agroclimatic and hydrogeological scores District-level maps illustrating the integrated score range (0–28 points) for agroclimatic and hydrogeological conditions from 2021 to 2024. All observed values fall within the unsatisfactory (0–13) or satisfactory (14–23) categories of the scoring system.

## Discussion

### The role of agroclimatic indicators in agricultural management

The set of agroclimatic indicators examined in this study collectively highlights the strong environmental constraints shaping agricultural productivity across the Jizzakh Region. Each parameter, radiation balance, VPD, aridity index, *C*_*m*_, *I*_*m*_, HTC, BCP, and ACPI, captures a different dimension of the regional hydrothermal regime, yet all converge on the same conclusion: the Jizzakh Region is experiencing persistent semi-arid conditions with a clear tendency toward increasing aridification, consistent with broader trends documented for Central Asia.

Radiation balance is a fundamental driver of ET, photosynthesis, and soil heat flux, and its role in shaping crop water demand has been consistently emphasized in agroclimatic literature.^7^^,^^23^^,^^24^^,^^25^ In the Jizzakh context, the classification into good, satisfactory, and unsatisfactory categories (240–270, 210–240, and 180–210 kJ/cm^2^) reflects the region’s variable but generally moderate solar energy supply. These radiation levels indicate that crops can achieve stable growth when irrigation and soil-moisture conservation techniques are properly managed. Nonetheless, limited radiation in some years, coupled with high evaporative demand, underscores the need for adaptive crop selection and enhanced soil management. The gradual increase in annual *R* values observed from 2021 to 2024 (approximately +3–6 kJ cm^−2^ month^−1^) may reflect regional warming trends and reduced cloud cover during the vegetation period.^26^ In practical terms, these results emphasize that districts with higher radiation balance require increased irrigation rates and careful water management, whereas areas with lower radiation balance present greater potential for water saving and resource-efficient agricultural practices. The VPD reinforces these findings. As a semi-arid region, Jizzakh experiences regular deficits that represent a major constraint on crop productivity. Based on FAO-56 conventions^24^ and regional agroclimatic practice, vegetation-period mean VPD values below ∼1.0 kPa indicate relatively favorable moisture conditions, values between 1.0 and 1.5 kPa indicate moderate atmospheric water demand, and values exceeding ∼1.5 kPa reflect high evaporative stress requiring full irrigation support. The aridity index further contextualizes regional moisture limitations. It should be noted that the radiation aridity index applied here follows the Budyko formulation based on radiation balance (*R*/*L*) rather than FAO reference ET (PET); therefore, values are not directly comparable with PET/*P* indices commonly reported in global studies but are fully consistent for intra-regional comparison. Numerous studies have documented the heightened sensitivity of Central Asian ecosystems to climatic variability, warming trends, and glacier retreat.^27^^,^^28^^,^^29^^,^^30^^,^^31^^,^^32^^,^^33^^,^^34^^,^^35^ Results from Jizzakh align with these regional observations, showing that climatic aridity is a structural feature of the landscape. Minor differences between irrigated and total areas suggest that existing irrigation systems effectively buffer climatic stress, stabilizing crop development under dry conditions. However, aridity values above 1.0 highlight rising water scarcity and signal the increasing importance of water-saving technologies and drought-resistant crops for long-term sustainability.

*C*_*m*_ and HTC provide complementary perspectives on the region’s hydrothermal regime. Persistently low *C*_*m*_ values emphasize the growing difficulty of meeting crop water requirements, especially during the vegetation period. The classification of *C*_*m*_ (>1.0, 0.6–1.0, and <0.6) reflects a shift toward moisture deficits that necessitate improved irrigation efficiency, soil moisture conservation, and crop diversification. HTC values (0.4–1.1) further characterize the region as semi-arid, corroborating earlier climatic zonations^36^^,^^37^ and confirming that annual fluctuations in rainfall and temperature produce unstable hydrothermal conditions. The observed variability in HTC also highlights the need for flexible, adaptive water management strategies capable of responding to sudden changes in moisture availability. *I*_*m*_, calculated using air humidity deficit rather than direct evaporation following the recommendations of Mustafayev et al.^7^^,^^22^ and Amanov,^38^^,^^39^ provides a refined indicator of atmospheric moisture conditions. Consistent with the patterns observed in *C*_*m*_ and HTC, *I*_*m*_ values confirm a progressive trend toward aridification, especially during the vegetation period. The classification (>1.0, 0.7–1.0, and <0.7) reveals that Jizzakh and Dustlik fall predominantly within the high-aridity range, indicating structural dependence on irrigation and heightened vulnerability to climatic fluctuations. BCP and meliorative soil bonitet (*B*_*m*_) assessments show that climate-driven anomalies, particularly changes in precipitation, directly influence productivity potential. These parameters are essential for understanding long-term agricultural sustainability under climate stress. Their integration allows the identification of areas requiring intensified soil rehabilitation or, alternatively, the introduction of water-saving technologies in high-fertility zones.^38^^,^^39^^,^^40^^,^^41^^,^^42^ The combined approach strengthens the scientific basis for land evaluation and supports targeted management strategies. Finally, the ACPI synthesizes key climatic variables, temperature and precipitation, into a single indicator of agricultural suitability. The ACPI values observed in Jizzakh correspond to desert and semi-desert categories according to Mustafayev et al.,^7^^,^^22^ and are consistent with classifications by Thornthwaite and Holtzman,^43^ Ivanov,^36^ and Selyaninov.^44^ When interpreted alongside *C*_*m*_, HTC, *I*_*m*_, BCP, and aridity indices, the ACPI confirms that water scarcity, drought risk, and climatic instability are long-standing and intensifying challenges for regional agriculture.

Taken together, the full set of climatic and meteorological indicators demonstrates a coherent pattern: the Jizzakh Region faces chronic water limitations and an increasingly unstable hydrothermal regime, exacerbated by broader climatic trends across Central Asia. These findings emphasize the need for integrated and adaptive water resource management, selection of drought-resistant crop varieties, application of water-saving technologies, and continuous monitoring of agroclimatic conditions to maintain agricultural productivity undergoing climate change.

### The role of soil and groundwater indicators in agricultural management and land reclamation

The assessment of soil and groundwater parameters reveals a set of interconnected hydrological constraints that continue to shape the reclamation status and productivity of irrigated lands in Jizzakh Region. Groundwater depth remains one of the most influential factors, as large portions of irrigated fields have historically exhibited groundwater levels shallower than 3 m, a threshold widely recognized as critical for the onset of soil waterlogging and secondary salinization. Observations from the 2021–2024 vegetation periods confirm that this vulnerability persists, presenting a spatially uneven pattern driven by differences in irrigation intensity, soil texture, drainage performance, and local climatic conditions. Comparisons with analogous patterns in Syrdarya Region, where groundwater commonly fluctuates between 1.5 and 2.5 m,^45^ indicate that groundwater in Jizzakh generally lies deeper; however, the gradual rise recorded in several districts still represents a reclamation concern. Elevated groundwater enhances capillary rise into the root zone, thereby increasing moisture during sensitive crop growth stages and accelerating salt accumulation, an effect extensively documented in earlier studies across Central Asia.^42^^,^^43^^,^^44^ Although groundwater typically recedes to depths below 2.5 m during February–May, creating favorable conditions for leaching and soil aeration, districts such as Sh. Rashidov show that this window is narrowing, necessitating additional measures to maintain groundwater depths consistent with automorphic soil regimes. Closely linked to groundwater depth is groundwater mineralization, which remains a central diagnostic indicator of salinization risk in arid irrigated regions. As widely reported for Central Asia, mineralization levels in groundwater, irrigation water, and soils tend to be elevated^45^^,^^46^^,^^47^ and are exacerbated by suboptimal irrigation and drainage management at the farm scale.^40^^,^^46^^,^^48^ The dominance of groundwater mineralization in the 1–3 g/L range, classified as moderately mineralized under the Komilov-Akhmedov system,^49^ is consistent with patterns observed in Central Fergana during 2018–2021,^50^ yet the expansion of 3–5 g/L zones in Jizzakh signals localized deterioration of hydro-reclamation conditions. Understanding mineralization thresholds is essential for determining critical groundwater depths, which vary by soil texture and moisture regime. For groundwater with 1–3 g/L mineralization (76.7% of the region in 2024), the critical depth ranges from 1.4 to 2.4 m in heavy soils to 1.2–1.3 m in light soils, whereas waters with 3–5 g/L mineralization (12.6% in 2024) require correspondingly deeper thresholds. These values align with recommendations for Jizzakh, including the use of a *C*_*m*_ of 0.12–0.22 to assess groundwater influence.^46^ Encouragingly, the area with low-mineralized groundwater (0–1 g/L) increased from 2.2% in 2023 to 9.9% in 2024, while strongly mineralized waters (>5 g/L) remain limited (<1%). Nevertheless, areas with moderate to high mineralization require strict control of groundwater depth to prevent rapid salinization.

The status of soil salinity reflects the cumulative impact of groundwater dynamics, mineralization, and irrigation practices. The continuing decline in saline and strongly saline soils over the past decade, attributed largely to the transition from furrow irrigation to water-saving technologies now implemented on more than 50% of irrigated land, demonstrates that irrigation modernization, coupled with reclamation interventions, is effectively mitigating salinity buildup. Even so, the persistence of salinity risks in areas with shallow groundwater or elevated mineralization underscores the need for continuous monitoring and targeted reclamation strategies. These relationships are further clarified by the water-salt balance, which integrates soil, agroclimatic, hydrological, and management factors to provide a comprehensive indicator of reclamation status. Variability across districts in irrigation inputs, precipitation, and drainage outflows strongly influences salt leaching and accumulation, consistent with established findings on water-salt dynamics.^42^^,^^51^^,^^52^^,^^53^ While precipitation contributes minimally to salinity due to its low mineral content, it plays a crucial role in promoting natural leaching during wet years. Total salt removal values suggest that reclamation efforts remain broadly effective, although districts exhibiting high water outflows highlight opportunities for improving irrigation efficiency and drainage performance. Positive trends in Mirzachul and Paxtakor illustrate the benefits of coherent reclamation management. Still, districts with water deficits or positive salt accumulation require enhanced interventions such as vertical drainage systems, controlled leaching operations, and precise irrigation scheduling. These outcomes reinforce long-standing conclusions on the importance of drainage and water management for preventing salinization.^16^^,^^47^^,^^54^^,^^55^^,^^56^^,^^57^^,^^58^^,^^59^

Taken together, the analysis of groundwater depth, mineralization, soil salinity, and water-salt balance confirms that the stability and productivity of irrigated landscapes in Jizzakh depend on maintaining a delicate hydrological equilibrium. Persistent risks of secondary salinization, driven mainly by shallow or mineralized groundwater, require continued investment in infrastructural and methodological solutions, and regular monitoring to safeguard long-term soil fertility and agricultural sustainability.

### Synthesis of the integrated assessment

The integrated assessment framework successfully combined agroclimatic, soil, groundwater, and water-salt balance indicators into a unified scoring system for evaluating the sustainability of irrigated agricultural landscapes in the Jizzakh Region. The district-level assessment yielded integrated scores ranging from 11 to 16 points during 2021–2024. Most districts were classified as unsatisfactory in 2021, whereas the majority attained satisfactory status during 2022–2024. However, scores generally remained within the lower portion of the satisfactory range, indicating that favorable conditions were not consistently achieved across all evaluated indicators. Considerable spatial and temporal variability was also observed among individual climatic, hydrogeological, and soil parameters. The results demonstrate that agroclimatic factors remain the principal constraints on agricultural productivity and landscape sustainability in the region. Elevated VPD, increasing climatic aridity, and low HTCs during dry years consistently reflected high atmospheric water demand and growing pressure on irrigated agriculture. At the same time, groundwater conditions and soil salinity generally remained within acceptable limits, although localized increases in groundwater mineralization and salinity risk highlight the continued need for effective drainage and reclamation measures.

The integrated assessment framework successfully combined agroclimatic, soil, groundwater, and water-salt balance indicators into a unified scoring system for evaluating the sustainability of irrigated agricultural landscapes in the Jizzakh Region. Although the overall classification indicates relatively stable reclamation conditions, with most studied areas classified as satisfactory during 2021–2024, considerable spatial and temporal variability was observed among individual indicators. The results demonstrate that agroclimatic factors remain the principal constraints on agricultural productivity and landscape sustainability in the region. Persistent moisture deficits, increasing climatic aridity, and low HTCs during dry years consistently reflected high atmospheric water demand and growing pressure on irrigated agriculture. At the same time, groundwater conditions and soil salinity generally remained within acceptable limits, although localized increases in groundwater mineralization and salinity risk highlight the continued need for effective drainage and reclamation measures. Districts with lower integrated scores, particularly Zafarabad and, during some years, Sh. Rashidov, may therefore require enhanced monitoring and targeted management interventions.

Beyond its application in the Jizzakh Region, the proposed framework provides a practical approach for integrating hydrological, soil, and climatic information within a single assessment system. The methodology facilitates comparison among districts, supports the early identification of vulnerable areas, and provides a quantitative basis for irrigation planning, salinity management, and land-reclamation activities. The district-level results suggest that management priorities differ across the region. For example, districts such as Zafarabad and, during some years, Sh. Rashidov, which achieved the lowest integrated scores, may benefit from enhanced monitoring of groundwater conditions, improved drainage maintenance, and targeted reclamation measures. In contrast, districts characterized by elevated aridity, high VPD, and low moisture-index values may require greater emphasis on irrigation scheduling, water-saving technologies, soil-moisture conservation practices, and the adoption of drought-tolerant cropping systems.^59^ Areas exhibiting increasing groundwater mineralization or persistent salinity risks would benefit from controlled leaching operations, involving the use of polymer-based biosolvents to enhance salt dissolution, and stricter management of groundwater depths to prevent secondary salinization.^60^ Because the assessment relies primarily on indicators already collected through existing hydrometeorological, groundwater-monitoring, and land-reclamation programs, it can be readily incorporated into existing regional monitoring systems without requiring substantial additional data collection. Furthermore, the three-level scoring scheme functions as a practical “traffic-light” system, allowing complex agroclimatic, soil, and groundwater information to be translated into a simple and easily interpretable management tool for farmers, water-management organizations, and regional authorities. The framework therefore provides a practical basis for prioritizing interventions, evaluating management effectiveness, and supporting climate-resilient agricultural development in irrigated dryland environments.

### Limitations of the study

Several limitations of this study should be acknowledged. The framework primarily relies on seasonal to annual agroclimatic indicators, which may not fully capture short-term extremes such as heat waves, episodic droughts, or irrigation disruptions that can critically affect crop performance. In addition, climatic conditions were characterized using two representative meteorological stations corresponding to the principal agricultural landscape zones of the Jizzakh Region. Although these stations provide the most complete and consistent long-term observations available for the study area, they may not fully capture local microclimatic variability, particularly in mountainous environments. Nevertheless, the use of long-term meteorological records and the focus on annual and seasonal agroclimatic indicators reduce the influence of short-scale spatial variability and provide a consistent basis for interannual comparisons. Also, groundwater depth and salinity assessments depend on monitoring networks with heterogeneous spatial coverage, potentially introducing local uncertainties in data-sparse areas. The use of finer spatial and temporal data would further improve diagnostic accuracy, and additional calibration is needed for different soil types, management regimes, and hydrological conditions. Another limitation is related to the integrated scoring procedure itself. To ensure transparency and comparability across indicators, all variables were incorporated into a unified scoring framework. However, the relative influence of climatic, soil, and hydrogeological factors may vary among regions and agricultural systems. Future research should therefore evaluate alternative weighting approaches and sensitivity analyses to further refine the assessment methodology.^61^^,^^62^^,^^63^^,^^64^ In addition, future developments could integrate higher-resolution remote sensing products, process-based crop and soil-salinity models, and more detailed management information to better resolve spatial and temporal variability and further enhance the robustness and applicability of the framework. Future research should also focus on validating the integrated scoring method in other regions and on the development of digital tools for real-time assessment and decision support.

Although the proposed framework was developed and validated for the Jizzakh Region, its conceptual structure is inherently transferable to other irrigated agroecosystems in arid and semi-arid environments. The framework relies on widely available climatic variables, established agroclimatic indices, and commonly monitored soil and groundwater parameters, enabling its application in regions characterized by strong atmospheric demand and irrigation-dependent agriculture. Nevertheless, regional calibration of indicator thresholds and scoring schemes is required to account for differences in soil texture, cropping systems, irrigation practices, groundwater dynamics, and climatic seasonality. In humid or predominantly rainfed systems, some components, particularly those related to groundwater salinity and water-salt balance may require modification or exclusion, indicating that the framework is most directly applicable to dryland and irrigated landscapes.

## Resource availability

### Lead contact

Requests for further information and resources should be directed to and will be fulfilled by the lead contact, Paolo Tarolli (paolo.tarolli@unipd.it).

### Materials availability

This study did not generate new unique material.

### Data and code availability

• Data reported in this paper will be shared by the lead contact upon request.
• This paper does not report original code.
• Any additional information required to reanalyze the data reported in this paper is available from the lead contact upon request.

## Acknowledgments

This study was carried out within the framework of the state-budget applied project no. 1.15 entitled “Enhancing the agro-resource potential of irrigated agro-landscapes and adapting water-saving technologies,” implemented by the Department of Use of Hydromeliorative Systems of the National Research University “Tashkent Institute of Irrigation and Agricultural Mechanization Engineers.” The research was supported by the Agritech National Research Center and received funding from the European Union 10.13039/100031478NextGenerationEU (PIANO NAZIONALE DI RIPRESA E RESILIENZA (PNRR) – MISSIONE 4 COMPONENTE 2, INVESTIMENTO 1.4 – D.D. 1032 17/06/2022, CN00000022). P. T. and A. Ghirardelli acknowledge the institutional support provided by TIIAME within the framework of ongoing scientific collaboration and academic exchange, including research visits conducted in May 2025.

## Author contributions

Conceptualization, B.A. and A.S.; methodology, B.A.; data curation, B.A., A. Gafarova, and D. G.; writing – original draft, B.A. and A. Ghirardelli; writing – review & editing, A. Ghirardelli and P.T.; visualization, A. Ghirardelli; validation, A. Ghirardelli and P.T.; software, A. Gafarova and D. G.; supervision, A.S. and P.T.

## Declaration of interests

P.T. is a member of the iScience Advisory Board.

## STAR★Methods

### Key resources table

**Table:** 

REAGENT or RESOURCE	SOURCE	IDENTIFIER
**Deposited data**		

Agro-meteorological measurements from weather stations of the Jizzakh region (Dustlik and Jizzakh stations)	Jizzakh Regional Hydrometeorological Center, Hydrometeorological Service Agency under the National Committee of Ecology and Climate Change of the Republic of Uzbekistan	https://gov.uz/oz/hydromet/departments/regional?active=3261
District-level agrometeorological variables	Syrdarya–Zarafshan Irrigation Systems Basin Department, Ministry of Water Resources of the Republic of Uzbekistan	https://gov.uz/en/suvchi/departments/regional?active=641
District-level irrigation water volumes, irrigated agricultural areas, inter-farm and on-farm irrigation network performance	Syrdarya–Zarafshan Irrigation Systems Basin Department, Ministry of Water Resources of the Republic of Uzbekistan	https://gov.uz/en/suvchi/departments/regional?active=641
Groundwater levels (annual average) for districts of the Jizzakh Region.	Syrdarya–Zarafshan Irrigation Systems Basin Department, Ministry of Water Resources of the Republic of Uzbekistan	https://gov.uz/en/suvchi/departments/regional?active=641
Groundwater mineralization by district of the Jizzakh Region.	State Fund for Geodesy-Cartography, Cadastre Agency of the Republic of Uzbekistan	https://gov.uz/en/kadastr/departments/subordinate?active=3950
Soil salinization level by district of the Jizzakh Region.	State Fund for Geodesy-Cartography, Cadastre Agency of the Republic of Uzbekistan	https://gov.uz/en/kadastr/departments/subordinate?active=3950

### Experimental model and study participant details

Omitted as our study does not involve biological models.

### Method details

#### Study area and data sources

The Jizzakh Region (southeast Uzbekistan) comprises 12 districts and the city of Jizzakh. The region covers 2,117,830 ha, of which 1,374,248 ha (64.9%) is agricultural land. Irrigated cropland occupies 515,372 ha, representing 24.3% of the region’s total area and 37.5% of agricultural land, while the remainder consists of non-irrigated cropland and rangeland. Land use follows a clear mountain–piedmont–plain gradient: rainfed and forage systems with extensive pastures dominate uplands and piedmont zones, whereas irrigated cotton, winter wheat, and horticultural production are concentrated on alluvial plains. The regional meteorological observation network includes stations located in Forish, Dustlik, Bakhmal, Gallaorol, and Jizzakh city.

Meteorological indicators, including air temperature, precipitation, relative humidity, wind speed, number of sunny days, geographic latitude, and elevation above Baltic Sea level, were collected from the “Dustlik” and “Jizzakh” meteorological stations, whose data were provided by the Jizzakh Regional Hydrometeorological Center for the years 2021–2024.^65^ The two stations were selected because of their broader parameter coverage, continuous long-term time series, and spatial representativeness based on the agricultural landscape classification of the Jizzakh Region. Based on Amanov et al.,^66^ dividing the territory into plain, piedmont, and alluvial zones, the Dustlik station represents the lowland irrigated plain conditions of the north area, whereas Jizzakh station reflects the climatic conditions of the central and piedmont-influenced part of the region. Since all the remaining agroclimatic variables were reported by the Syrdarya-Zarafshan Irrigation Systems Basin Department (SZISBD)^67^ at the district level, each district was aligned with the corresponding agricultural landscape zone for subsequent computation analysis. Specifically, the Arnasoy, Dustlik, Mirzachul, Paxtakor and Zafarobod districts were assigned to the Dustlik station, while the Sharof Rashidov district, Zarbdor, Zomin districts were assigned to the Jizzakh station. Although these two stations cannot fully capture all local microclimatic variations, particularly in mountain landscapes, they were used to characterize the general agroclimatic background of the irrigated agricultural districts. Moreover, climatic indicators were combined with district-level soil salinity, groundwater depth, groundwater mineralization, and water-salt balance indicators for a more comprehensive assessment.

Information on groundwater levels of the Jizzakh Region was obtained from the annual reports of the Melioration Expedition under SZISBD,^67^ providing monthly and annual data at the district level. 2016 was also included for baseline comparison with the years 2021–2024. The aggregated district-level incidence of groundwater mineralization and soil salinity in irrigated lands was sourced from the State Fund for Geodesy-Cartography.^68^ District-level irrigation water volumes and irrigated agricultural areas for the 2021–2024 irrigation seasons were obtained from the Ministry of Water Resources and SZISBD.^67^ The irrigation season was defined as April 1–October 30, while the non-vegetation period from November to March was additionally considered where leaching-related diagnostics were required. Information on inter-farm and on-farm irrigation networks (irrigation and collector-drainage water quality, irrigation and drainage network condition, and drainage performance) was also collected at the district level.

#### Agroclimatic indicators

##### Net radiation (R)

Evapotranspiration primarily depends on the radiation balance, with solar radiation serving as the main energy source. The net radiation (R) was calculated following the FAO methodology^23^^,^^24^:

(Equation 1) $$R=R_{s}-R_{l}$$

where *R* is net radiation (kJ·cm^−2^·month^−1^), *R*_*s*_ represents incoming shortwave radiation (kJ·cm^−2^·month^−1^), and *R*_*l*_ denotes outgoing longwave radiation (kJ·cm^−2^·month^−1^).

##### Vapor Pressure Deficit (*VPD*)

The vapor pressure deficit (*VPD*) was determined in accordance with FAO-56 guidelines^24^:

(Equation 2) $$VPD=e_{s}-e_{a}$$

where *e*_*s*_ is the saturated vapor pressure (kPa), and *e*_*a*_ is the actual vapor pressure (kPa). Monthly and annual values were derived from maximum and minimum air temperatures, and the results were aggregated seasonally and annually to assess variations in atmospheric moisture demand.

##### Aridity index

The aridity index characterizes the relationship between available energy and atmospheric moisture supply^69^:

(Equation 3) $$\bar{R}=\frac{R}{L\cdot P}$$

where *R*is the net radiation (kJ·cm^−2^), *L* is the latent heat of evaporation (kJ·kg^−1^), and *P* is precipitation (mm).

Besides the classic equation, a modified Budyko’s equation, accounting for both rainfed and irrigated agricultural conditions, was applied to calculate the aridity index for the entire area^66^:

(Equation 4) $${\bar{R}}_{UM}=\frac{R}{L\cdot \left(P+V+V_{H}+V_{V}+F+\nabla W_{a}\right)}$$

Where *V* the total volume of water supplied to the area, $V_{H}$ and $V_{V}$ the water used for irrigation from horizontal and vertical collector–drainage networks, respectively, *F* the filtration losses in main canals, and *∇W*_*a*_ the changes in water reserves within the aeration zone.

For irrigated areas only, an additional modified Budyko’s equation, incorporating irrigation input and groundwater exchange processes, was used^66^:

(Equation 5) $${\bar{R}}_{CM}=\frac{R}{L\cdot \left(P+I_{r}+V_{H}+V_{V}+\left(1-\alpha \right)F_{K}+\nabla W_{a}\pm g^{a}\right)}$$

Where *Iᵣ* is the volume of water supplied directly to irrigated areas, *α* the proportion of groundwater recharge through canal filtration, *F*_*k*_ the seepage losses of water through canal beds, and *g*^*a*^ the water exchange between the aeration zone and groundwater.

##### Natural moisture coefficient

The natural moisture coefficient (*C*_m_) expresses the balance between precipitation and potential evaporation^36^:

(Equation 6) $$C_{m}=\frac{P}{0.0018{\left(25+T\right)}^{2}\left(100-f\right)}$$

where *P* is precipitation (mm), *T* is average monthly temperature (°C), and *f* is relative humidity (%).

##### Moisture index

The moisture index ($I_{m}$) quantifies the relationship between precipitation and the humidity deficit^6^:

(Equation 7) $$I_{m}=\frac{P}{\sum d}$$

where *∑d* is the vapor pressure deficit (kPa).

##### Hydrothermal coefficient (HTC)

The *HTC* evaluates the balance between heat and moisture availability during the growing season^19^:

(Equation 8) $$HTC=\frac{10P}{\Sigma T_{>10{}^{\circ}C}}$$

where *P* is total precipitation during periods with average daily temperature above 10°C (mm), and *Σ* $T_{>10{}^{\circ}C}$ is the sum of daily temperatures above 10°C (°C).

##### Biological–Climatic Productivity index (*BCP*)

The *BCP* evaluates potential agricultural productivity as a function of temperature and moisture conditions^6^:

(Equation 9) $$BCP=C_{m}\cdot \frac{\sum T}{1000}$$

Where *C*_*m*_ is the moisture coefficient, and ∑T is the sum of temperatures for vegetation or non-vegetation periods. Calculations were performed separately for vegetation period (defined as the part of the year when average daily temperature exceeds 10°C) and non-vegetation period (the remaining part of the year) to capture seasonal differences in productivity potential.

##### Meliorative Soil Bonitet (B_m_)

An integrated indicator, the Meliorative Soil Bonitet (B_m_), quantitatively assessing soil fertility and productive capacity, and providing a standardized measure to classify soils according to their suitability for agriculture, has been recently developed in Uzbekistan to combine biological productivity with soil salinity effects^39^:

(Equation 10) $$B_{m}=B_{\max}\left(1-S_{r}\right)\frac{Y_{act}}{Y_{\max}}F_{h}$$

where *B*_*max*_ is the maximum biological–climatic productivity value, $S_{r}$ is the proportion of saline area relative to total land, $Y_{act}$ and $Y_{\max}$ represent actual and potential yields respectively, and $F_{h}$ accounts for humus content, agrotechnical level, and soil salinization status. An increase in $S_{r}$ proportionally reduces *B*_*m*_, reflecting deterioration of soil quality and reclamation status.

##### Agroclimatic potential index (*ACPI*)

The agroclimatic potential index (*ACPI*) was calculated according to Mustafayev and Ryabtsev^7^:

(Equation 11) ACPI=P/5.12T+306

where coefficients 5, 12, and 306 are empirical constants derived from Mustafayev and Ryabtsev.^7^

#### Soil and groundwater indicators

The soil and groundwater status of irrigated lands was evaluated through a suite of hydrological and soil indicators reflecting the intensity of salinization processes and the balance of water and salts within the root zone. Core variables included groundwater level and mineralization, soil salinity, and the overall water–salt balance. Calculations followed the classical balance method developed by Averyanov et al.^54^^,^^55^^,^^56^ with modifications to account for the irrigation–drainage context of the Jizzakh Region. Methodological elements from Ikramov et al.^70^ were also incorporated. To ensure comparability and local relevance, all parameters and coefficients were calibrated to regional hydrogeological and climatic conditions using empirical data from monitoring wells and irrigation system records.

##### General water–salt balance

The overall water–salt balance for the irrigated area, where *ΔW* denotes the change in water reserves within the balance contour (m^3^·ha^−1^) and *ΔS* represents the change in salt reserves within the same contour (t·ha^−1^) was determined using two specific equations. The water balance was expressed as:

(Equation 12) $$\Delta W=P+I-ET-Q_{d}-D_{h}-D_{v}$$

where *P* is precipitation input, *I* is the volume of irrigation water supplied, *ET* is evapotranspiration loss, $Q_{d}$ represents total water discharge from irrigated fields and technical canal losses, $D_{h}$ denotes drainage outflow through horizontal systems (calculated according to Yakubov et al.^47^), and $D_{v}$ is water withdrawn through vertical drainage wells (based on operational data from pumping stations).

The salt balance was determined by:

(Equation 13) $$\Delta S=S_{P}+S_{I}-S_{D}-S_{leach}$$

where $S_{P},S_{I},S_{D}$, and $S_{leach}$ are the respective salt fluxes (t·ha^−1^) associated with precipitation, irrigation water, drainage discharge, and leaching processes.

The ratio between inflow and outflow components was adjusted using empirical relationships derived from experimental and production-scale studies.^58^ These formulations build upon FAO-56 guidelines^2^^,^^24^ and regional approaches that explicitly incorporate irrigation–drainage interactions.^38^ All calculations were calibrated using local meteorological, hydrological, and soil quality datasets to ensure representativeness under the conditions of the Jizzakh Region.

##### Groundwater and soil parameters

The groundwater table (GWT, m) was analyzed as district average value. Groundwater mineralization (GWM, g·L^−1^) and Soil salinity level (SSL) were expressed as district-level percentages. Soil classes were based on dry residue of soluble salts and chlorine content in the 0–100 cm soil layer, with increasing classes reflecting greater salt accumulation, as proposed by Ikramov,^71^ Khamidov and Matyakubov.^46^^,^^47^^,^^71^

#### Quality control of climate and hydrogeological indicators and consistency assessment

All input data were subjected to quality control, including range checks, outlier screening, unit harmonization, and spatial-temporal consistency checks before being incorporated into the integrated assessment. Moreover, all climatic and hydrogeological indicators were computed on an annual basis and compared with long-term reference values from the Jizzakh (1936–2024) and Dustlik (1973–2024) meteorological stations. This comparison allowed the identification of interannual variability and deviations from historical climatic norms, providing context for assessing their effects on soil and groundwater conditions. To ensure consistency and validation of the groundwater and soil quality parameters, groundwater levels measured between 2008 and 2011 at 30 observation wells in the Bobur and Yangiobod areas of the neighboring Syrdarya Region, were analyzed and compared to the Jizzakh dataset to ensure consistency and validation of the soil quality parameters. The wells were installed by the Japan International Research Center for Agricultural Sciences (JIRCAS), in collaboration with the Farmers’ Council of Uzbekistan and the Ministry of Agriculture and Water Resources.^45^

The selected indicators collectively describe the hydrothermal regime, water–energy balance, and soil–groundwater interactions that determine the stability and productivity of irrigated agroecosystems in the region. Climatic parameters were derived from station-based meteorological records, whereas soil and groundwater quality indicators were obtained from systematic monitoring of groundwater levels, mineralization, and soil salinity.

### Quantification and statistical analysis

To set up an Integrated Scoring-Based assessment of climatic, and hydrogeological conditions, calculations were based on the classical balance method developed by Averyanov et al.,^54^^,^^55^^,^^56^ with adaptations to the irrigation–drainage context of the Jizzakh Region. Methodological elements from Ikramov et al.^70^ were also incorporated. To ensure comparability and local relevance, all parameters and coefficients were calibrated to regional hydro-geological and climatic status using empirical data from monitoring wells and irrigation-system records.

To enable an integrated evaluation of the studied parameters, all climatic, soil, and groundwater quality indicators were normalized and classified following a unified procedure. For each indicator, monthly and seasonal series for the period 2021–2024 were compiled and analyzed. Threshold values were established using a combination of empirical data distributions and accepted agroclimatic, hydrogeological, and land-reclamation criteria. For climatic indicators, the observed range of values was divided into three equal intervals corresponding to unfavorable, intermediate, and favorable conditions, which were assigned scores of 0, 1, and 2, respectively. Groundwater and soil indicators were classified according to established threshold values commonly used in reclamation and irrigation^71^^,^^72^^,^^73^ and subsequently assigned to the same three-level scoring scheme. The directionality of each variable was taken into account: parameters for which higher values indicate stress or degradation (groundwater mineralization, soil salinity, vapor pressure deficit, and aridity) were scored inversely, whereas indicators representing favorable hydrothermal or agroclimatic conditions (water balance, radiation balance, biological–climatic productivity, agroclimatic potential, melioration soil bonitet, moisture index, hydrothermal coefficient, and groundwater table depth) were scored directly (Table S7).

The resulting partial scores were summed to obtain a composite index (maximum = 28 points) describing the overall agroclimatic and hydrogeological condition of irrigated landscapes. According to the total score, areas were classified as *Good* (24–28 points), *Satisfactory* (14–23 points), or *Unsatisfactory* (0–13 points, less than 50% of the total points). This integrated scoring system provides a quantitative and spatially consistent framework for evaluating the agroclimatic, and hydrogeological status of agricultural landscapes. By combining meteorological, soil, and groundwater indicators within a single analytical structure, the approach enables rapid comparison among districts and landscape zones and supports both scientific assessment and practical decision-making for sustainable land and water management under changing climatic and hydrological conditions.
