# Supplementary material for: An integrated framework for agroclimatic, salinity, and groundwater assessment in the Jizzakh Region, Uzbekistan
Source: iScience. 2026 Oct 6;29(11):117582. doi: 10.1016/j.isci.2026.117582 (PMC13676131; doi:10.1016/j.isci.2026.117582)
Supplement: Supplementary file 1 [file mmc1.pdf]

iScience, Volume *29*

Supplemental information

An integrated framework for agroclimatic, salinity, and groundwater assessment in the Jizzakh Region, Uzbekistan

B. Amanov, A. Ghirardelli, A. Sherov, A. Gafarova, D. Gulomov, and P. Tarolli

Table S1. Radiation balance (*R*, kJ cm^{-2} month^{-1}) of the vegetation and non-vegetation period for total and irrigated areas by district of Jizzakh Region, 2021–2024.

Year	District	Total area (non-veget.)	Total area (veget.)	Irrig. area (non-veget.)	Irrig. area (veget.)
2021	Arnasay	121	182	122	186
2022		122	182	124	186
2023		124	177	125	181
2024		124	182	125	186
2021	Dustlik	122	180	124	184
2022		122	177	124	182
2023		124	176	125	181
2024		124	182	125	186
2021	Zafarabad	122	178	124	185
2022		122	176	124	182
2023		123	175	125	181
2024		123	180	125	186
2021	Mirzachul	122	179	124	184
2022		122	177	124	182
2023		124	177	125	181
2024		123	181	125	186
2021	Paxtakor	122	180	124	185
2022		122	177	124	182
2023		124	176	125	181
2024		124	181	125	186
2021	Sh.Rashidov	107	174	109	179
2022		99	175	100	180
2023		110	176	111	181
2024		125	186	147	211
2021	Zarbdor	107	174	109	179
2022		99	174	100	180
2023		110	176	111	181
2024		145	205	147	211
2021	Zomin	106	172	109	180
2022		98	173	100	180
2023		109	174	111	182
2023		109	174	111	182
2024		144	202	147	212

Table S2. District-level Aridity Index (classic Budyko's equation, $\overline{R}$) for total and irrigated areas in Jizzakh Region, 2021–2024. Values are given separately for the vegetation and non-vegetation periods.

Year	District	Total area			Irrigated area		
		Non-vegetation	Vegetation	Annual	Non-vegetation	Vegetation	Annual
2021	Arnasay	0.21	2.35	0.47	0.22	2.40	0.48
2022		0.17	0.54	0.29	0.18	0.56	0.30
2023		0.24	1.18	0.45	0.24	1.20	0.46
2024		0.24	0.46	0.33	0.24	0.47	0.34
2021	Dustlik	0.22	2.32	0.47	0.22	2.38	0.48
2022		0.17	0.53	0.29	0.17	0.55	0.29
2023		0.24	1.17	0.45	0.24	1.21	0.46
2024		0.24	0.46	0.33	0.24	0.47	0.34
2021	Zafarabad	0.22	2.30	0.47	0.22	2.38	0.48
2022		0.17	0.53	0.29	0.17	0.55	0.29
2023		0.24	1.16	0.44	0.24	1.21	0.46
2024		0.24	0.46	0.33	0.24	0.47	0.34
2021	Mirzachul	0.22	2.31	0.47	0.22	2.38	0.48
2022		0.17	0.53	0.29	0.17	0.55	0.29
2023		0.24	1.18	0.45	0.24	1.20	0.46
2024		0.24	0.46	0.33	0.24	0.47	0.34
2021	Paxtakor	0.22	2.32	0.47	0.22	2.38	0.48
2022		0.17	0.53	0.29	0.17	0.55	0.29
2023		0.24	1.17	0.45	0.24	1.21	0.46
2024		0.24	0.46	0.33	0.24	0.47	0.34
2021	Sh.Rashidov	0.19	1.82	0.43	0.20	1.88	0.44
2022		0.14	0.28	0.21	0.14	0.29	0.21
2023		0.18	1.15	0.37	0.18	1.19	0.38
2024		0.28	0.53	0.38	0.28	0.54	0.39
2021	Zarbdor	0.19	1.82	0.43	0.20	1.88	0.44
2022		0.14	0.28	0.21	0.14	0.29	0.21
2023		0.18	1.15	0.37	0.18	1.19	0.38
2024		0.28	0.52	0.38	0.28	0.54	0.39
2021	Zomin	0.19	1.80	0.43	0.20	1.88	0.44
2022		0.14	0.28	0.20	0.14	0.29	0.21
2023		0.18	1.14	0.37	0.18	1.19	0.38
2024		0.27	0.52	0.38	0.28	0.54	0.39

Table S3. District-level Aridity Index (modified Budyko's equation) for total ($\bar{R}_{UM}$) and irrigated ($\bar{R}_{CM}$) areas in Jizzakh Region, 2021–2024. Values are given separately for the vegetation and non-vegetation periods.

Year	District	Total area			Irrigated area		
		Non-vegetation	Vegetation	Total	Non-vegetation	Vegetation	Total
2021	Arnasay	1.19	2.84	1.83	1.59	1.34	1.43
2022		1.25	1.54	1.41	1.56	0.96	1.14
2023		1.67	2.00	1.85	2.08	0.89	1.16
2024		1.92	1.49	1.64	2.43	0.95	1.26
2021	Dustlik	0.79	2.11	1.26	1.31	1.39	1.36
2022		1.23	1.33	1.29	1.76	1.18	1.36
2023		1.15	1.68	1.41	1.71	1.70	1.71
2024		1.42	1.56	1.50	2.04	1.63	1.77
2021	Zafarabad	1.04	1.94	1.44	1.87	1.39	1.55
2022		1.20	1.39	1.30	1.88	1.26	1.45
2023		1.23	1.66	1.45	2.22	1.23	1.51
2024		1.72	1.30	1.44	2.78	1.21	1.56
2021	Mirzachul	0.80	2.11	1.27	1.57	1.46	1.50
2022		1.21	1.49	1.36	1.78	1.28	1.45
2023		1.39	1.22	1.29	2.20	1.17	1.45
2024		1.24	1.18	1.21	2.03	1.23	1.46
2021	Paxtakor	1.14	1.60	1.37	1.69	1.38	1.49
2022		0.65	1.56	0.99	0.84	1.34	1.08
2023		1.24	1.87	1.55	2.12	1.11	1.38
2024		1.73	1.37	1.50	2.67	1.03	1.37
2021	Sh.Rashidov	0.95	1.49	1.23	1.45	1.50	1.48
2022		0.98	1.08	1.04	1.53	1.35	1.41
2023		1.10	1.29	1.21	1.29	1.25	1.26
2024		1.57	1.26	1.37	1.72	1.38	1.50
2021	Zarbdor	0.90	1.50	1.20	1.24	1.29	1.27
2022		0.88	1.07	0.99	1.01	1.19	1.12
2023		1.29	1.44	1.38	1.54	1.15	1.27
2024		1.88	1.41	1.57	2.08	1.31	1.54
2021	Zomin	1.00	1.32	1.17	1.42	6.62	2.79
2022		1.21	0.93	1.02	2.44	4.46	3.44
2023		1.31	1.28	1.29	1.69	4.51	2.76
2024		1.80	1.27	1.44	2.28	3.37	2.81

Table S4. Comparative analysis of hydrothermal coefficient (HTC) values at Dustlik and Jizzakh meteorological stations for 2021–2024 and long-term averages.

Meteorological station	2021	2022	2023	2024	Long-term average
**Jizzakh**	0.40	1.09	0.46	0.77	0.76 (90 y)
**Dustlik**	0.42	0.95	0.41	0.84	0.66 (52 y)

Table S5. Water-salt balance inputs in the districts of Jizzakh region (2021–2024), including irrigation, precipitation, and salt loading.

District	Years	Irrigation water (m^3/ha)	Precipitation (m^3/ha)	Salt via irrigation (t/ha)	Total water (m^3/ha)	Total salt (t/ha)
Arnasay	2021	7882	2567	6.16	10449	6.16
	2022	7870	4166	6.8	12036	6.8
	2023	7971	2688	7.25	10659	7.25
	2024	7408	3648	6.72	11056	6.72
Dustlik	2021	10076	2567	8.63	12643	8.63
	2022	7127	4166	5.71	11293	5.71
	2023	7802	2688	6.62	10490	6.62
	2024	6078	3648	5.13	9726	5.13
Zafarabad	2021	9190	2567	12.24	11757	12.24
	2022	7626	4166	6.47	11792	6.47
	2023	8882	2688	9.69	11570	9.69
	2024	7419	3648	8.02	11067	8.02
Mirzachul	2021	10110	2567	8.91	13037	8.91
	2022	6927	4166	5.69	11453	5.69
	2023	7502	2688	6.01	10550	6.01
	2024	7000	3648	5.62	11008	5.62
Paxtakor	2021	8744	2567	11.19	11311	11.19
	2022	11071	4166	9.25	15237	9.25
	2023	8320	2688	7.31	11008	7.31
	2024	7258	3648	6.36	10906	6.36
Sh.Rashidov	2021	9182	2795	7.4	11977	7.4
	2022	7554	5303	6.27	12857	6.27
	2023	8245	3061	6.6	11306	6.6
	2024	7905	3674	6.33	11579	6.33
Zarbdor	2021	8816	2795	6.71	11611	6.71
	2022	7052	5303	5.57	12355	5.57
	2023	7549	3061	6.87	10610	6.87
	2024	7182	3674	6.53	10856	6.53
Zomin	2021	8046	2795	6.34	10841	6.34
	2022	6811	5303	5.72	12114	5.72
	2023	6538	3061	5.68	9599	5.68
	2024	6799	3674	5.91	10473	5.91

Table S6. Output components of the water-salt balance in the districts of Jizzakh region (2021–2024), showing evapotranspiration, drainage, and salt removal.

District	Years	ET (m³/ha)	Total drainage (m³/ha)	Salt removed (t/ha)	Total water (m³/ha)	Total salt (t/ha)
Arnasay	2021	8588	2053	2.05	13256	2.05
	2022	8790	1747	2.44	13360	2.44
	2023	9092	1737	2.59	13672	2.59
	2024	8888	1677	2.4	13208	2.4
Dustlik	2021	8352	4695	2.44	13047	2.44
	2022	7965	3263	1.61	11228	1.61
	2023	6274	3781	2.39	10054	2.39
	2024	6344	3110	1.83	9453	1.83
Zafarabad	2021	8860	4957	3.46	13817	3.46
	2022	8833	4208	1.83	13040	1.83
	2023	8875	4769	2.74	13644	2.74
	2024	8524	3958	2.26	12483	2.26
Mirzachul	2021	8279	5600	2.46	13879	2.46
	2022	8079	3721	1.81	11800	1.81
	2023	8351	2444	0.59	10795	0.59
	2024	8061	2727	0.55	10788	0.55
Paxtakor	2021	7449	4199	3.06	11647	3.06
	2022	8766	6746	2.53	15511	2.53
	2023	8871	4517	2	13387	2
	2024	8929	3880	1.74	12809	1.74
Sh.Rashidov	2021	8564	3898	2.02	12462	2.02
	2022	8981	4432	1.71	13413	1.71
	2023	8843	3095	1.81	11942	1.81
	2024	8545	3002	1.73	11552	1.73
Zarbdor	2021	7671	3745	1.84	11417	1.84
	2022	7995	3859	1.6	11854	1.6
	2023	8175	3225	1.97	11399	1.97
	2024	8093	3124	1.88	11217	1.88
Zomin	2021	8072	3871	1.9	11942	1.9
	2022	7803	7003	1.71	14805	1.71
	2023	7515	3218	1.7	10734	1.7
	2024	7604	3146	1.77	10750	1.77

Table S7. Summary of indicators, units, threshold values, and scoring criteria used in the integrated assessment.

Indicator	Value range	Score (points)	Interpretation
Radiation balance (R, kJ cm^{-2} year^{-1})	280–306	2	Good
	>306–308	1	Average
	>308–359	0	Bad
Aridity index ($\bar{R}$, dimensionless)	0.21–0.34	2	Good / less arid condition
	>0.34–0.44	1	Average / moderate aridity
	>0.44–0.48	0	Bad / high aridity
Modified aridity index for irrigated area ($\bar{R}_{CM}$, dimensionless)[a]	1.08–1.37	2	Good / less arid condition
	>1.37–1.50	1	Average / moderate aridity
	>1.50–3.44	0	Bad / high aridity
Vapor Pressure Deficit (VPD, kPa)	0.89–1.01	2	Good / low atmospheric moisture deficit
	>1.01–1.16	1	Average / moderate atmospheric moisture deficit
	>1.16–1.27	0	Bad / high atmospheric moisture deficit
Natural moisture coefficient (C_m, dimensionless)	0.13–0.16	0	Bad / low natural moisture supply
	>0.16–0.33	1	Average / moderate natural moisture supply
	>0.33–0.47	2	Good / relatively higher natural moisture supply
Moisture index (I_m, dimensionless)	0.06–0.07	0	Bad / low moisture condition
	>0.07–0.17	1	Average / moderate moisture condition
	>0.17–0.21	2	Good / relatively favorable moisture condition
Hydrothermal coefficient (HTC, dimensionless)	0.40–0.42	0	Bad / low hydrothermal moisture condition
	>0.42–0.84	1	Average / moderate hydrothermal moisture condition
	>0.84–1.09	2	Good / favorable hydrothermal moisture condition
Biological–climatic productivity (BCP, dimensionless)	0.66–0.94	0	Bad / low biological–climatic productivity
	>0.94–1.70	1	Average / moderate biological–climatic productivity
	>1.70–2.43	2	Good / high biological–climatic productivity
Melioration soil bonitet (MSB, dimensionless)	0.63–1.26	0	Bad / poor and below-average lands
	>1.26–1.89	1	Average / medium-quality lands
	>1.89–3.15	2	Good / good and best-quality lands
Agroclimatic Potential Index (ACPI, dimensionless)	0.04–0.05	0	Bad / low agroclimatic potential
	>0.05–0.08	1	Average / moderate agroclimatic potential
	>0.08–0.11	2	Good / high agroclimatic potential
Water balance (ΔW, m³ ha^{-1})	-361 − +361	2	Good / balanced water regime
	<-1135 − -361 or >+361 − +1135	1	Average / moderately unbalanced water regime
	<−1135 or >+1135	0	Bad / strongly unbalanced water regime
Groundwater table depth (m below ground surface)	1–2 m	0	Bad / shallow groundwater table
	2–3 m	1	Average / moderately shallow groundwater table
	>3 m	2	Good / deep and safer groundwater table
Groundwater mineralization (g L^{-1})	0–1	2	Good / fresh groundwater
	>1–3	1	Average / slightly mineralized groundwater
	>3	0	Bad / moderately to strongly mineralized groundwater
Salt balance (ΔS, t ha^{-1})	-8.39 − +0.66	2	Good / salt removal or very low salt accumulation
	+0.66 − +1.68	1	Average / moderate salt accumulation
	>+1.68 − +6.50	0	Bad / high salt accumulation risk
Soil salinity level (SSL) [b]	Non-saline or slightly saline	2	Good / low salinity risk
	Moderately saline	1	Average / moderate salinity risk
	Strongly or very strongly saline	0	Bad / high salinity risk

[a]The modified equation was used in place of the standard $\bar{R}$ for irrigated land. [b]Soil salinity classification based on dry residue of soluble salts and chlorine content in the 0–100 cm soil layer. Non-saline: <0.3% dry residue and <0.01% chlorine; slightly saline: 0.3–1.0% dry residue and 0.01–0.05% chlorine; moderately saline: 1.0–2.0% dry residue and 0.05–0.10% chlorine; strongly saline: 2.0–3.0% dry residue and 0.10–0.15% chlorine; very strongly saline: >3.0% dry residue and >0.15% chlorine.

Table S8. District-level integrated score describing the agroclimatic and hydrogeological state of the agricultural landscape, years 2021-2024.

District	years	Total score	Agricultural landscape status
Arnasay	2021	12	Unsatisfactory
	2022	16	Satisfactory
	2023	14	Satisfactory
	2024	16	Satisfactory
Dustlik	2021	12	Unsatisfactory
	2022	16	Satisfactory
	2023	15	Satisfactory
	2024	15	Satisfactory
Zafarabad	2021	11	Unsatisfactory
	2022	15	Satisfactory
	2023	13	Unsatisfactory
	2024	15	Satisfactory
Mirzachul	2021	13	Unsatisfactory
	2022	16	Satisfactory
	2023	15	Satisfactory
	2024	16	Satisfactory
Pakhtakor	2021	12	Unsatisfactory
	2022	15	Satisfactory
	2023	15	Satisfactory
	2024	16	Satisfactory
Sh.Rashidov	2021	12	Unsatisfactory
	2022	16	Satisfactory
	2023	13	Unsatisfactory
	2024	14	Satisfactory
Zarbdor	2021	12	Unatisfactory
	2022	14	Satisfactory
	2023	14	Satisfactory
	2024	15	Satisfactory
Zomin	2021	12	Unsatisfactory
	2022	15	Satisfactory
	2023	15	Satisfactory
	2024	16	Satisfactory
